# BiT age: A transcriptome‐based aging clock near the theoretical limit of accuracy

**DOI:** 10.1111/acel.13320

**Published:** 2021-03-03

**Authors:** David H. Meyer, Björn Schumacher

**Affiliations:** ^1^ Institute for Genome Stability in Ageing and Disease Medical Faculty University of Cologne Cologne Germany; ^2^ Cologne Excellence Cluster for Cellular Stress Responses in Ageing‐Associated Diseases (CECAD) Center for Molecular Medicine Cologne (CMMC) University of Cologne Cologne Germany

**Keywords:** aging, aging clock, biological aging, biomarkers, *Caenorhabditis elegans*, RNA sequencing, transcriptome

## Abstract

Aging clocks dissociate biological from chronological age. The estimation of biological age is important for identifying gerontogenes and assessing environmental, nutritional, or therapeutic impacts on the aging process. Recently, methylation markers were shown to allow estimation of biological age based on age‐dependent somatic epigenetic alterations. However, DNA methylation is absent in some species such as *Caenorhabditis elegans* and it remains unclear whether and how the epigenetic clocks affect gene expression. Aging clocks based on transcriptomes have suffered from considerable variation in the data and relatively low accuracy. Here, we devised an approach that uses temporal scaling and binarization of *C. elegans* transcriptomes to define a gene set that predicts biological age with an accuracy that is close to the theoretical limit. Our model accurately predicts the longevity effects of diverse strains, treatments, and conditions. The involved genes support a role of specific transcription factors as well as innate immunity and neuronal signaling in the regulation of the aging process. We show that this binarized transcriptomic aging (BiT age) clock can also be applied to human age prediction with high accuracy. The BiT age clock could therefore find wide application in genetic, nutritional, environmental, and therapeutic interventions in the aging process.

## Graphical abstract

Using *Caenorhabditis elegans* RNA seq and lifespan datasets we developed a binarized transcriptomic aging (BiT age) clock with a precision close to the theoretical limit of accuracy that can also be applied to humans. The BiT age clock allows quantification of pro‐ and anti‐aging effects of genetic, nutritional, environmental and pharmacological interventions in the aging process.

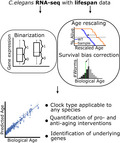

## 1 INTRODUCTION

Aging is the driving factor for several diseases, the declining organ function, and overall progressive loss of physiological integrity. Aging biomarkers that predict the biological age of an organism are important for identifying genetic and environmental factors that influence the aging process and for accelerating studies examining potential rejuvenating treatments. Diverse studies tried to identify biomarkers and predict the age of individuals, ranging from proteomics, transcriptomics, the microbiome, frailty index assessments to neuroimaging, and DNA methylation (Galkin et al., 2020). Currently, the most common predictors are based on DNA methylation. The DNA methylation marks themselves might influence the transcriptional response, but aging also affects the transcriptional network by altering the histone abundance, histone modifications, and the 3D organization of chromatin. The difference in RNA molecule abundance, thereby, integrates a variety of regulation and influences resulting in a notable gene expression change during the lifespan of an organism (Lai et al., 2019). These changes sparked interest in the identification of transcriptomic aging biomarkers, an RNA expression signature for age classification, and the development of transcriptomic aging clocks.

Peters et al. extended previous classification approaches to a regression, which allows the computation of the predicted age and developed a transcriptional aging clock based on whole‐blood microarray samples for half of the human genome and reported an *r* ^2^ of up to 0.6, an average difference of 7.8 years, and an association of the predicted age to blood pressure as well as smoking status (Peters et al., 2015). Similarly, Mamoshina et al. build a transcriptomic aging clock of human muscle tissue. A deep feature selection model performed best with an *r* ^2^ of 0.83 and a mean absolute error of 6.24 years (Mamoshina et al., 2018). However, microarray data have the drawbacks of a limited range of detection, high background levels, and the detection of just a subset of the transcriptome. Instead, by applying an ensemble of linear discriminant analysis classifiers on RNA‐seq data, a model with an *r* ^2^ of 0.81, a mean absolute error of 7.7 years, and a median absolute error of 4.0 years were obtained in a dataset derived from cell culture of healthy donors (Fleischer et al., 2018). The same model also predicted an accelerated age in 10 patients with the premature aging disease Hutchinson‐Gilford progeria syndrome (HGPS).

While a large variety of data, techniques, and analyses have been used to identify aging biomarkers and aging clocks in humans, issues remain with regard to pronounced variability and difficulties in replicability. Indeed, a recent analysis of gene expression, plasma protein, blood metabolite, blood cytokine, microbiome, and clinical marker data showed that individual age slopes diverged among the participants over the longitudinal measurement time and subsequently that individuals have different molecular aging pattern, called ageotypes (Ahadi et al., 2020). These interindividual differences show that it is still difficult to pinpoint biomarkers for aging in humans.

Model organisms, instead, can give a more controllable view on the aging process and biomarker discovery. *Caenorhabditis elegans* has revolutionized the aging field and has vast advantages as a model organism. Even isogenic nematodes in precisely controlled homogenous environments have surprisingly diverse lifespans; however, the underlying causes are still incompletely understood. Several predictive biomarkers of *C. elegans* aging have been described, and a first transcriptomic clock of *C. elegans* aging using microarray data of 104 single wild‐type worms predicted the chronological age with 71% accuracy (Golden et al., 2008). When the prediction was based on modular genetic subnetworks inferred from microarray data with support vector regression, the age of sterile *fer*‐*15* mutants at 4 timepoints was predicted with an *r* ^2^ of 0.91. The same approach on the 104 individual N2 wild‐type worms yielded an *r* ^2^ of 0.77 indicating that for microarray data subnetworks of genes result in better prediction compared with single gene predictors, likely due to the noisiness of the data type (Fortney et al., 2010). Although the accuracy of this model is reasonable, it is limited by the fact that no lifespan‐affecting genotypes or treatments were tested and that the validation dataset, although tested on single worms, resulted in an increased prediction error. Recently, an initial age prediction based on microarray data predicted 60 RNA‐seq samples with a Pearson correlation of 0.54 and was improved to an r of 0.86 when the chronological age was rescaled by the median lifespan of the corresponding sample (Tarkhov et al., 2019). Even though this model instead of chronological age predicted the biological age of a variety of *C. elegans* genotypes, it is limited by the accuracy of the prediction. Moreover, the biological age is not reported in days, but as a variable with values between 0 and ~2.5, which makes it harder to interpret.

To date, no aging clock for *C. elegans* has been built solely on RNA‐seq data and been shown to predict the biological age of diverse strains, treatments, and conditions to a high accuracy. In this study, we build such a transcriptomic aging clock that predicts the biological age of *C. elegans* based on high‐throughput gene expression data to an unprecedented accuracy. We combine a temporal rescaling approach, to make samples of diverse lifespans comparable, with a novel binarization approach, which overcomes current limitations in the prediction of the biological age. Moreover, we show that the model accurately predicts the effects of several lifespan‐affecting factors such as insulin‐like signaling, a dysregulated miRNA regulation, the effect of an epigenetic mark, translational efficiency, dietary restriction, heat stress, pathogen exposure, the diet‐, and dosage‐dependent effects of drugs. This combination of rescaling and binarization of gene expression data therefore allows for the first time to build an accurate aging clock that predicts the biological age regardless of the genotype or treatment. Lastly, we show how our binarized transcriptomic aging (BiT age) clock model has the potential to improve the prediction of the transcriptomic age of humans and might therefore be universally applicable to assess biological age.

## 2 RESULTS

### 2.1 Temporal scaling and transcriptome data binarization allow precise biological clock predictions

**FIGURE 1:**
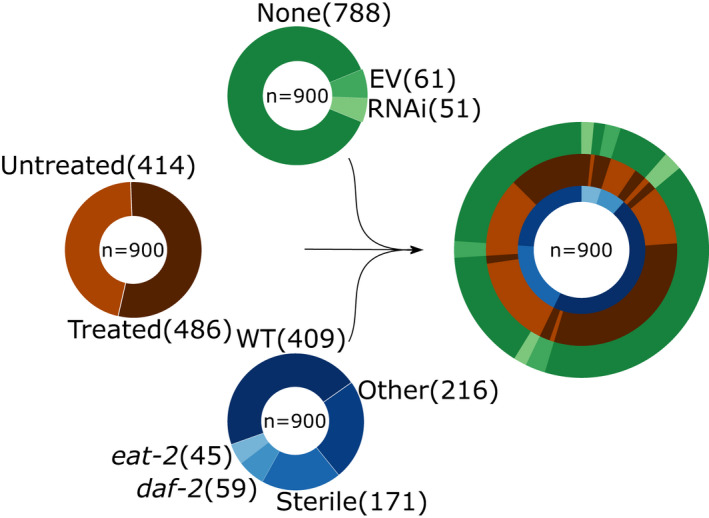
Data overview. Overview of the processed published data utilized in the training of the model. Pie charts show the distribution of different genotypes (blue), treatments (brown), and RNAis (green). The convoluted pie chart on the right shows the overlap of the three classes. The partition “Sterile” contains multiple different genotypes that cannot give rise to progeny and *daf*‐*2*, as well as *eat*‐*2*, might contain additional mutations. For a more detailed view, see Table S1

We downloaded and processed 1,020 publicly available RNA‐seq samples for adult *C. elegans* out of which for 972 samples corresponding lifespan data were available (Table S1). 900 samples were used for the training and testing of the model, the remainder for validation purposes (Figure 1). Out of the 900 samples most (409) were wild‐type N2 worm populations. A significant portion of 171 samples contained reads of temperature‐sensitive sterile strains such as *glp*‐*1* or *fem*‐*1* or double mutants thereof. 59 samples contained a mutation in the insulin‐like growth factor 1 receptor *daf*‐*2* and 45 a mutation in the dietary‐restriction mimic strain *eat*‐*2* either as a single or as a combination with a different mutation. 216 samples did not cluster in one of the mentioned groups and contain a variety of different strains. 112 of the samples span 14 different RNAis in 51 samples and 61 empty vector controls. Slightly more than half of the samples (486) were sequenced from a population that was undergoing a treatment (excluding RNAi or empty vector) that is different from the standard treatment of an *Escherichia coli* OP50 diet at 20°C. The convoluted circle plot on the right side of Figure 1 shows the overlap of the different possible combinations of strains, RNAi, and treatments in our training samples.

We only downloaded and processed data for which the corresponding publication reported a median lifespan. The lifespan data are required to make strains with vastly different lifespans comparable. Without rescaling, an RNA‐seq sample of a long‐lived nematode beyond the normal lifespan of a wild‐type worm would not be comparable to a wild‐type sample, since no sample would be able to be generated. Lifespan‐altering manipulations, for example, a temperature shift, a *daf*‐*2* mutation, or oxidative damage, were shown to just shift the lifespan curve by stretching or shrinking it (Stroustrup et al., 2016). One interpretation would be that all lifespan‐affecting interventions converge on similar pathways, which affect the risk of death in a similar pattern, just at different velocities. Moreover, there have been descriptions of a transcriptional drift during *C. elegans* aging (Hastings et al., 2019; Tarkhov et al., 2019), which might be due to a (dys‐)regulation of single transcription factors (Mann et al., 2016) and the suppression of this transcriptional drift might slow down the aging process (Rangaraju et al., 2015). Notably, age prediction could be improved by rescaling the chronological age by the median lifespan (Tarkhov et al., 2019).

We, therefore, employed a strategy similar to Tarkhov et al. and rescaled the lifespan by the corresponding median lifespan of the sample. We set the median lifespan of a standard wild‐type N2 worm to µ = 15.5 days of adulthood. Using this standard lifespan, we calculated a correction factor to determine the biological age of a sample. For example, the correction factor of a strain with a measured median lifespan of 31 days would be µ/31 = 0.5 and thereby assuming a uniform aging rate reduction of 50%. This correction factor would be applied to each RNA‐seq sample of the same strain and experiment. A sample sequenced, for example, at day 10 of adulthood, would be corrected to 10*0.5 = 5 days of biological age. Applying the individual correction factors for each RNA‐seq sample allows us to build a classifier of the biological, instead of the chronological age. Importantly, by defining a standard lifespan of 15.5 days we are able to predict the biological age in days instead of a variable between 0 and 2.5 as reported by Tarkhov et al.

Owing to the fact that the public data were generated in multiple laboratories with different protocols and sequencers (see Table S1 for details), we expected noisy data with a strong batch effect. Indeed, the results of an elastic net regression (see Methods for details) on the raw counts‐per‐million (CPM) reads resulted in a mediocre model with an *r* ^2^ of 0.78, a Pearson correlation of 0.89 (*p* = 2.82e‐304), a Spearman correlation of 0.86 (p = 9.97e‐258), a mean absolute error (MAE) of 1.02 days, a median absolute deviation (MAD) of 0.71 days, and a root‐mean‐square‐error (RMSE) of 1.51 days. Figure S1a shows the comparison of the rescaled biological age of the strains on the *x*‐axis and the age predicted by the elastic net regression on the y‐axis. Interestingly, the overall absolute error and the variance in the absolute error of the prediction increase strongly after ~5 days (Figure S2).

In order to mitigate this increase in variance, we developed a novel approach and binarized the transcriptome data by setting the value of each gene to 1, if the CPM is bigger than the median CPM of the corresponding sample and 0 otherwise (see Methods for details), thereby reducing the noise, but retaining the information whether a gene is strongly transcribed or not. After this binarization, we trained an elastic net regression model with nested cross‐validation to obtain the best parameter setting and optimal set of genes (see Methods for details) that predict the biological age remarkably well with an *r* ^2^ of 0.96, a Pearson correlation of 0.98 (*p*<1e‐304), a Spearman correlation of 0.96 (*p*<1e‐304), a mean absolute error of 0.46 days, a median absolute error of 0.33 days, and a RMSE of 0.66 days (Figure S1b).

Interestingly, especially the increased variance in older samples, as seen in our initial analysis in Figure S1a, diminished and showed a strong improvement in overall accuracy. Comparison of the absolute error terms of the raw CPM and the binarized data prediction shows that the absolute error of the binarized prediction is lower than the prediction based on the raw CPMs regardless of the biological age of the worms. Furthermore, while the initial prediction on the raw data starts to get especially inaccurate starting from day 5, the increase in the binarized data is far less pronounced (Figure S2a). Interestingly, also the variance of the absolute error terms stays more stable in the binarized data than the raw data and thereby demonstrating a more robust prediction regardless of the true age of the worms (Figure S2b).

These results show that the binarization approach strongly improves the prediction, especially in older samples, which have been shown to contain a noisier transcriptome. Indeed, this age‐dependent noisiness so far hindered the identification of proper aging biomarkers. The binarization therefore might facilitate the identification by reducing the noise, while retaining the important information. To verify our prediction further, eight independent datasets, not used in the nested cross‐validation for optimization of the parameter and gene set, were predicted with an *r* ^2^ of 0.91, a Pearson correlation of 0.97 (*p* = 2.43e‐58), a Spearman correlation of 0.91 (*p* = 6.58e‐38), a mean error of 0.92 d, a median error of 0.53 d, and a RMSE of 1.40 d (Figure S1c).

The results show that the overall prediction is highly accurate; however, although lower than the increase in deviation in the raw data, the binarized data as well show a decrease in accuracy in samples with an older biological age (see also Figure S2). This might be due to the lower sample size of older animals, but might also be influenced by the nature of bulk RNA sequencing itself. Figure S3a shows a standard lifespan curve of *C. elegans*. Until ~day 8, 100% of non‐censored worms are alive. Starting from day 8, the first worms die, until the median lifespan is reached at ~15.5 days and the maximum at ~24 days. We can assume that the biological age of worms at the same chronological age follows a normal distribution (Figure S3b). In other words, in a plate of synchronized worms at day 8 we would expect to see that most worms are also at a biological age of 8 days. However, some worms will be healthier while others are already close to death and will therefore be the worms that start dying early. While the peak of this bell curve will therefore be the chronological age of the worm population, some worms will be biologically younger and some older (Figure S3b). Starting from the next day, the first part of the worm population will die (Figure S3c). Assuming the normal distribution of the biological age of the worms and a hypothetical maximum biological age as shown with the dotted line in Figure S3d, we can hypothesize that the biologically older worms will die off first and thereby truncate the biological age distribution on the right side of the curve (Figure S3d). This truncation will shift the true median biological age toward the left side, as indicated by the green line. This becomes more noticeable at the median lifespan of 15.5 days, where by definition 50% of the population is dead (Figure S3e). Following the same reasoning from above, we see that the right half of the biologically older worms died, while the younger half of the population stayed alive. However, this clearly skews the distribution, since the oldest 50% of the population is dead and therefore will not contribute to the average biological age anymore. Indeed, the median biological age will be the median of the remaining, alive worms, that is, the left part of the curve. This will result in a shift of biological age, especially for chronologically older populations (Figure S3f). In consideration of this biological age shift, an RNA‐seq sample sequenced at 15.5 days will have a younger true population‐median biological age, which will introduce a bias into the regression model. The bias will be not as pronounced in younger samples, since most of the population will still be alive (Figure S3b).

**FIGURE 2:**
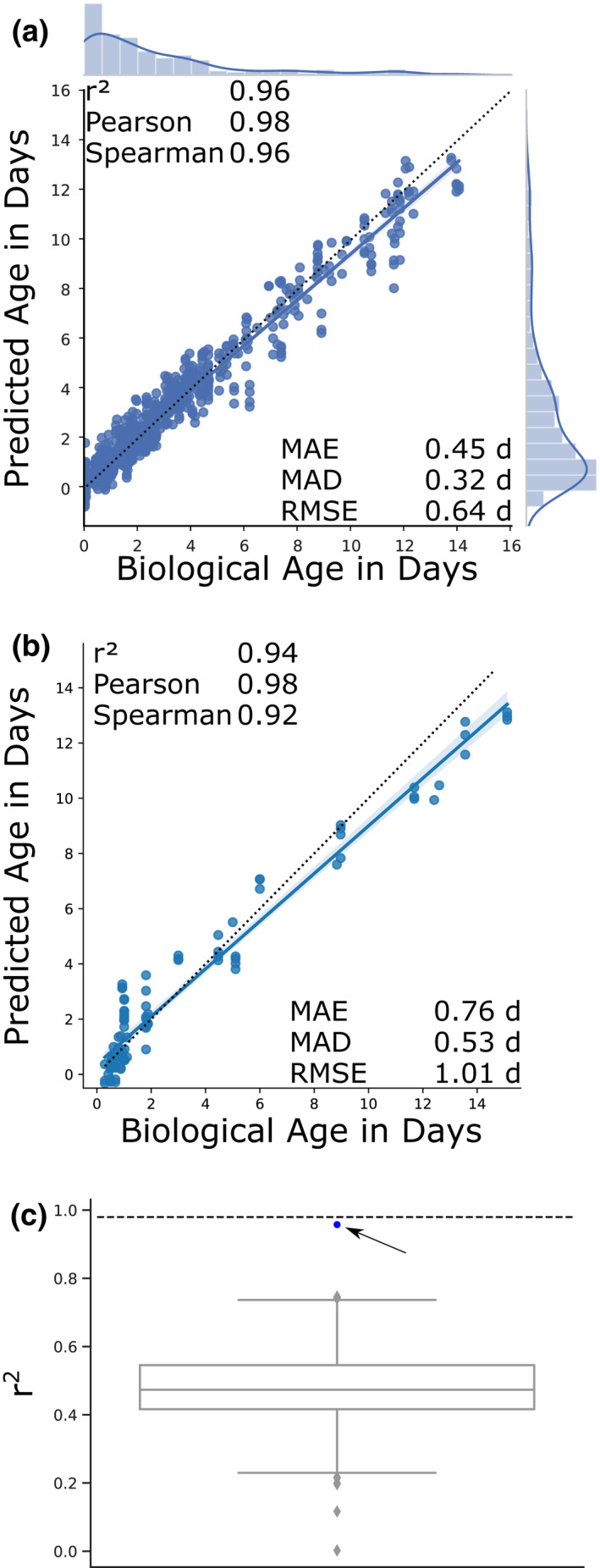
Biological age prediction. (a) Results of the biological age prediction computed by cross‐validation. The *x*‐axis shows the rescaled biological age in days starting from adulthood additionally corrected by the second rescaling approach. The *y*‐axis shows the predicted age computed by the elastic net regression after the second rescaling approach on binarized gene expression data. Every blue dot displays one RNA‐seq sample. The regression line with the 95% confidence interval is shown in blue, and the dotted line shows the perfect linear correlation. The distribution of the data is shown on the side of the plot. *r* ^2^ = coefficient of determination, Pearson = Pearson correlation, Spearman = Spearman correlation, MAE = mean absolute error in days, MAD = median absolute deviation in days, RMSE = root‐mean‐square‐error in days. (b) Prediction of the model on eight independent datasets consisting of 94 samples at different time points. The *x*‐axis shows the rescaled biological age in days starting from adulthood additionally corrected by the second rescaling approach. The *y*‐axis shows the predicted age computed by the elastic net regression after the second rescaling approach on binarized gene expression data. For more details on the data, see Table S1. (c) The *y*‐axis shows the *r* ^2^ of a given prediction. The box plot displays 1,000 random models with 576 genes. The prediction by our final model with an *r* ^2^ of 0.96 is shown as a blue dot and indicated by the arrow. The dotted line shows the theoretical limit of prediction given by the limit of accuracy in the chronological age annotation as well as variance in the lifespan data used for rescaling

To alleviate this bias, we calculated a second correction term that takes into consideration the hypothetical biological age distribution of the sequenced population (methods for details). Applying this correction before the optimization of the regression resulted in an improved prediction model, especially for the independent dataset. The new model utilizes 576 genes (Table S2) and predicts the full dataset slightly better, with an *r* ^2^ of 0.96, a Pearson correlation of 0.98 (*p*<1e‐304), a Spearman correlation of 0.96 (*p*<1e‐304), a mean error of 0.45 d (−1.63% compared with pre‐correction model), a median error of 0.32 d (−2.15%), and a RMSE of 0.64 d (−3.47%) (Figure 2a). The independent dataset is now predicted with an *r* ^2^ of 0.94, a Pearson correlation of 0.98 (*p* = 1.13e‐62), a Spearman correlation of 0.92 (*p* = 6.24e‐38), a mean error of 0.76 d (−17.45%), a median error of 0.53 d, and a RMSE of 1.01 (−28.28%) (Figure 2b). These data indicate that it might be worthwhile including a correction for the survival bias of worms in older populations. The comparison to the prediction on the unbinarized validation data after applying the second correction term showed a strong improvement in accuracy upon binarization with a 48.27% reduction in the mean error (Figure S4a, Table S3).

To confirm that not every gene set of 576 genes results in a similar prediction, we randomly sampled 576 genes and recorded the resulting absolute errors and *r* ^2^ values. The boxplot in Figure 2c shows the distribution of *r* ^2^ values centering around the mean of 0.488 with a standard deviation of 0.117. The blue dot shows the result of our predicted gene set as a clear outlier at 0.96. The MAE and MAD are centered around 1.27 d and 0.911 d with a standard deviation of 0.066 and 0.063, respectively (Figure S4b).

To assess the precision of the age prediction, we next probed how close this model approaches the theoretical limit of a biological clock. The datasets are annotated in whole days alive from adulthood and thereby including a variance of ±12 h to the actual chronological age. Random sampling of this error alone gives a mean error of 0.236 (±0.006) d, a median error of 0.187 (±0.006) d, and a *r* ^2^ of 0.986 (±0.002). However, since lifespan assays, even done under the same conditions in the same laboratory, will vary, we can assume that the reported median lifespan, used for the temporal rescaling, will also be including an inherent experimental error. Indeed, it has been shown that lifespan assays are heavily affected by the number of animals and less, but substantially, by the scoring frequency, thereby indicating that many lifespan studies are underpowered and often driven by stochastic variation (Petrascheck & Miller, 2017). Computing the mean and SD of lifespan assays for one genotype with the same treatment for several publications shows that the variation is indeed on average ~7% for one standard deviation from the mean with a range between 5.44% and 8.83% (Table S3). An assumption of a moderate 5% deviation between assays increases the mean error to 0.302 (±0.007) d, the median error to 0.244 (±0.008) d, and reduces the *r* ^2^ to 0.98 (±0.002). These theoretical optima, shown as dotted lines in the boxplots in Figure 2c and Figure S4b, clearly display the quality of our prediction. We conclude that the prediction based on the set of 576 genes is close to the theoretical optimum.

Next, we compared our model to a previous model (Tarkhov et al., 2019) that described three sets of aging‐associated genes. The first set, consisting of 327 genes, was generated by a meta‐analysis of publicly available microarray data, the second consists of 902 age‐associated genes generated by the analysis of 60 RNA‐seq samples, and finally, a sparse subset with only 71 genes that Tarkhov et al. used for their biological age prediction. The gene set derived from microarray data performed worst on the prediction of the 900 RNA‐seq samples with an *r* ^2^ of 0.52 and a mean error of 1.33 d (195.18% increase compared with our final model). The gene set of 902 genes performed similarly, with an *r* ^2^ of 0.57 and a mean error of 1.40 d (210.37% increase). Finally, the sparse predictor provided an *r* ^2^ of 0.57 and a mean error of 1.36 d (202.07% increase) (Figure S5a–c; for further quality measurements, see Table S3). Remarkably, binarization improves the prediction of these three gene sets as well to an *r* ^2^ of 0.74, 0.78, and 0.62, respectively (Figure S5d,e, Table S3). Although the *r* ^2^ of the sparse predictor increased to 0.62, the MAE and MAD increased and thereby also show that a single quality assessment is not enough to give a good evaluation (Figure S5f).

Next, we also evaluated the prediction of the independent datasets from Figure 2b with the three previously published gene sets. The gene set of 71 genes performed worst with an *r* ^2^ of 0.35 and a MAE of 1.95 d (+156.07% compared with our final model). The gene set derived from microarray data and the gene set with 902 genes performed better with an *r* ^2^ of 0.44 and a MAE of 2.20 d (+188.11%), respectively, an *r* ^2^ of 0.43 and a MAE of 2.31 d (+203.24%) (Figure S6a–c; for further quality measurements, see Table S3). Remarkably, the binarization could also improve the prediction in this case to an *r* ^2^ of 0.87 for the gene set derived from microarray data, 0.85 for the gene set of 902 genes, and 0.72 for the sparse predictor (Figure S6d–f; for further quality measurements, see Table S3).

These comparisons indicate that binarization is improving the quality of regression models overall and that our new model consisting of 576 binarized genes predicts the biological age of *C. elegans* to a high accuracy and superior to previously existing models.

### 2.2 Transcriptomic clock correctly predicts multiple lifespan‐affecting factors

Since our model is able to predict the biological age to a high accuracy, we next tested the capability of the model to predict the effect of multiple lifespan‐affecting factors. We used the previously determined 576 predictor genes and trained an elastic net regression on the 900 RNA‐seq samples, excluding the data for the respective publication. This is thereby a different cross‐validation approach where we excluded a whole experimental dataset at a time.

**FIGURE 3:**
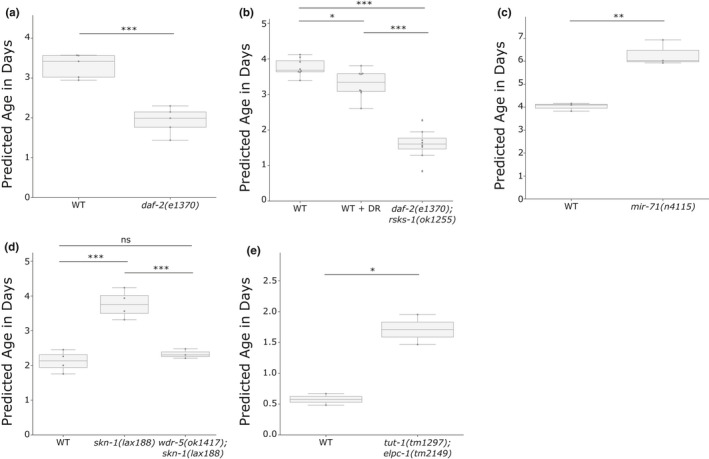
Biological age prediction of short‐ and long‐lived mutants. The box plots show the predicted biological age in days on the *y*‐axis. Assuming the properties of a uniform temporal rescaling, a lower predicted age will equal a longer lifespan. The corresponding whole dataset was set aside for the training of the final model for the corresponding plot. Blue dots display single RNA‐seq samples. (a) The lifespan‐extending *daf‐2(e1370)* strain is predicted to be biologically younger than WT samples of the same chronological age (4.5 days). Note that the WT strain in this publication had a longer lifespan (19.4 days) than the standard 15.5 days and is thereby also predicted to be biologically younger than its chronological age. Data from GSE36041. (b) Dietary restriction (DR) and the long‐lived double mutant *daf‐2(e1370)*; *rsks‐1(ok1255)* are predicted to be significantly younger than WT samples of the same chronological age (4 days). Data from GSE119485. (c) The lifespan‐shortening *mir‐71(n4115)* mutation significantly increased the predicted biological age compared to samples of the same chronological age (5 days). Data from GSE72232. (d) The gain‐of‐function mutant *skn‐1(lax188)* significantly increased the biological age, while an additional mutation in the epigenetic regulator *wdr‐5* rescues the biological age back to WT levels (2 days). Data from GSE123531. (e) The double mutant *tut‐1(tm1297)*; *elpc‐1(tm2149)* significantly increases the biological age (chronological age of 1 day). Data from GSE67387. *p < 0.05, **p ≤ 0.01, ***p ≤ 0.001, independent two‐sided *t* tests were used for comparisons in (a), (c), and (e). One‐way ANOVA with a post hoc Tukey test was used in (b) and (d). Table S3 contains more detailed statistics

First, we tested the well‐known effect of insulin‐like signaling on the biological age and saw that a *daf‐2* mutation reduces the predicted biological age compared with the WT strain of the same experiment by 41.3% in 4‐day adult *C. elegans* (Figure 3a). The even longer‐lived *daf‐2*; *rsks‐1* double mutant is accordingly predicted to be even younger with a significant reduction of 56.8% in 4‐day adults (Figure 3b).

To determine whether short‐lived mutants can also be predicted correctly, we next tested *mir‐71*, which has been shown to regulate the global miRNA abundance during aging and to directly influence lifespan (Inukai et al., 2018). Compared to WT, *mir‐71* mutants are predicted to be 56% older in 5‐day adults (Figure 3c). In addition, samples of a gain‐of‐function *skn‐1* mutation, that is, detrimental for lifespan, are predicted to be 77.2% older than wild‐type worms at day 2 (Figure 3d). Interestingly, this adverse effect can be rescued by a loss‐of‐function mutation in *wdr‐5* and the subsequent abolishment of the epigenetic mark H3K4me3 (Nhan et al., 2019), which is remarkably also reflected in our prediction. Loss of protein homeostasis decreases overall fitness and is a hallmark of aging. In *C. elegans*, the loss of uridine U34 2‐thiolation in *tut‐1*; *elpc‐1* double mutants has been shown to have a negative impact on the efficiency of translation and to promote protein aggregation (Nedialkova & Leidel, 2015). Strikingly, this effect on translational efficiency is also reflected in the transcriptomic aging clock for day 1 adults, which are predicted to be 196% older than their wild‐type counterpart (Figure 3e).

These data show that the BiT age clock can effectively predict the biological age of a variety of mutants and pathways, ranging from the insulin pathway, miRNAs, and the epigenetic mark H3K4me3 to translational efficiency.

Since both, long‐lived and short‐lived strains, are predicted with the correct pattern, we next asked whether we could predict the effect of dietary restriction (DR) on the biological age. Although the effect was slight, the dietary‐restricted worms are predicted to be 12.9% younger than their normal‐fed counterpart at day 4 of adulthood (Figure 3b). DR‐induced longevity was shown to depend on the PMK‐1/p38 signaling‐regulated innate immune response. In *C. elegans*, *sek‐1* is part of the PMK‐1/p38 signaling cascade and required for longevity in dietary‐restricted worms (Wu et al., 2019). Noticeably, the same trend can be observed in our prediction for day 6 adults (Figure S7a). A two‐way ANOVA showed a significant interaction between the effects of the strain and dietary restriction (*p* = 0.004), which indicates that the effect of DR is dependent on *sek‐1* activation. Although in this dataset, the adjusted p‐value of the effect of DR in WT worms is not significant (*p* = 0.057), it is interesting to note that the dietary‐restricted worms are on average 32% younger than the *ad libitum* fed WT worms. This biological age reduction is thereby showing a stronger effect than the 12.9% reduction in Figure 3b. This could be due to strain differences in the different laboratories or suggest that positive effects of DR add up over time.

**FIGURE 4:**
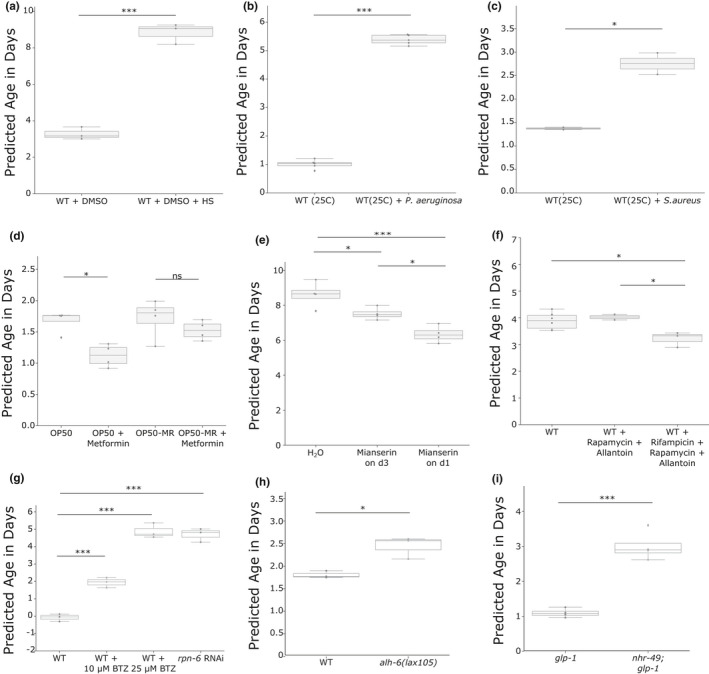
Biological age prediction of a variety of treatments and stressors. The box plots show the predicted biological age in days on the y‐axis. Assuming the properties of a uniform temporal rescaling, a lower predicted age will equal a longer lifespan. The corresponding whole dataset was set aside for the training of the final model for the corresponding plot. Blue dots display single RNA‐seq samples. (a) Heat shock induces a strong increase in the predicted biological age at a chronological age of 3 days in WT. Data from PRJNA523315. (b) Pathogen infection by *Pseudomonas aeruginosa* at 25°C at a chronological age of day 1 increases significantly the predicted age. Data from GSE122544. (c) Pathogen infection by *S. aureus* at 25°C at a chronological age of day 1 increases significantly the predicted age. Data from GSE57739. (d) The bacterial strain‐dependent effect of metformin is resembled in the prediction. The box plots show wild‐type worm populations at a chronological age of day 2 with either a standard OP50 *E. coli* diet or a Metformin‐resistant OP50 (OP50‐MR) strain with or without 50 mM Metformin. A two‐way ANOVA showed a significant treatment effect (*p* = 0.004). Data from E‐MTAB‐7272. (e) The dosage‐dependent effect of Mianserin is resembled in the prediction. The box plots show wild‐type worm populations at a chronological age of day 10 either treated with water or 50 µM Mianserin on day 3 or day 1. A one‐way ANOVA showed significance (*p* = 0.0008). Data from GSE63528. (f) The effect of drug combinations at the chronological age of 6 days is resembled in the prediction. A one‐way ANOVA showed significance (*p* = 0.02). Data from GSE108263. (g) An independent dataset without a reported lifespan sequenced at the chronological age of day 1. Wild‐type worms were treated with either 10 µM or 20 µM of the proteasome inhibitor Bortezomib (BTZ), or RNAi against the proteasomal subunit *rpn*‐*6*. Data from GSE124178. (h) An independent dataset without a reported lifespan sequenced at the chronological age of day 3. Data from GSE121920. The predicted median lifespan reduction of 35.7% is similar to the reported lifespan reduction of 33.5% (Pang & Curran, 2014). (i) An independent dataset without a reported lifespan sequenced at the chronological age of day 2. Data from GSE158729. The predicted median lifespan reduction of 63.96% is similar to the reported lifespan reduction of 50%–60.69% (Ratnappan et al., 2014). *p < 0.05, **p ≤ 0.01, ***p ≤ 0.001, independent two‐sided *t* tests were used for comparisons in (a), (b), (c), (h), and (i). One‐way ANOVA with a post hoc Tukey test was used in (e), (f), and (g). Two‐way ANOVA with a post hoc Tukey test was used in (d). Table S3 contains more detailed statistics

Next, we decided to test whether different lifespan‐shortening stressors can be predicted correctly. Both heat stress (Figure 4a) and pathogen exposure to either *P. aeruginosa* or *S. aureus* (Figure 4b,c) showed a strong increase in the predicted biological age. Heat stress increased the prediction by 169.3% in day 3 adults. *Pseudomonas aeruginosa* increased the predicted age by 421.4%. And *S. aureus* increased the biological age prediction by 101%, in day 1 adults.

While heat or pathogen exposure can lead to a quick demise of the animals, we wondered whether more subtle changes in lifespan by different diets and subsequent nutrient metabolism could also be detected. It was shown that an *E. coli* K12 variant's indole secretion extends fecundity and overall healthspan and lifespan in *C. elegans*, while an isogenic *E. coli* strain (K12tnaA) with a deletion in the indole‐converting gene does not have these benefits. This effect on healthspan was reported to be not yet visible in worms on day 8, but showed a significant difference only at the next tested timepoint on day 15 (Sonowal et al., 2017). Intriguingly, the same pattern can be observed in RNA‐seq samples of day 3 and day 12 (Figure S7b). A two‐way ANOVA showed a significant treatment effect (*p* = 0.034) indicating the sensitivity of the approach. Moreover, in accordance with the published results, a subsequent post hoc Tukey test showed no difference between the diets on day 3 (adjusted *p* = 0.9), while day 12 showed a 15.3% increased biological age in the K12tnaA diet (adjusted *p* = 0.0506). Consistent with the link between diet‐dependent changes in nutrient metabolism and lifespan, it has been shown that the lifespan‐extending effect of Metformin is, at least partially, regulated by a bacterial nutrient pathway (Pryor et al., 2019). A two‐way ANOVA of the predicted biological age of day‐2 adults, grown on either *E. coli* OP50 or a Metformin‐resistant OP50 strain, with or without Metformin showed as well a significant bacteria effect (*p* = 0.045) as a significant drug effect (*p* = 0.004). A subsequent post hoc Tukey test showed a significant reduction in the biological age of Metformin‐treated wild‐type worms grown on OP50 (−34.5%), but no significant effect in worms grown on Metformin‐resistant OP50 (Figure 4d).

Next, we asked whether the effect of the duration time of a drug might be reflected on the transcriptomic age. The antidepressant Mianserin has been shown to extend the lifespan of *C. elegans* by inhibiting serotonergic signals, which is lessening the age‐dependent transcriptional drift. This effect is more pronounced in animals that were treated starting from day 1, compared to starting the treatment from day 3 (Rangaraju et al., 2015). Our prediction of day 10 adults resembles this conclusion; a one‐way ANOVA showed a significant difference (*p* = 0.0008) and an ensuing post hoc Tukey test revealed statistical significance between all three cases, with the biggest effect in worms treated from day 1 (Figure 4e).

An interesting and challenging question is whether the combination of different lifespan‐extending drugs might have a synergistic effect. Admasu et al. reported that not all combinations of drugs have an additive effect. While the combination of Rapamycin with Allantoin had no effect on the lifespan of wild‐type worms, the triple combination with Rifampicin surprisingly had the biggest effect (Admasu et al., 2018). Interestingly, while the administration of rifampicin, rapamycin, and allantoin significantly reduced the predicted age by 17.7% (Figure 4f), the double combination of rapamycin and allantoin did not change the predicted lifespan, which is in accordance with the published lifespan results.

Lastly, we decided to check the biological age prediction of independent validation data and downloaded three datasets for which no direct lifespan data (i.e., in the same publication) were published and which contained treatments and strains that were not included in any of the analyses and nested cross‐validations above. We first tested the effect of proteotoxic stress on the transcriptional age with samples of two different dosages of the proteasome inhibitor bortezomib (BTZ) and the knockdown by RNAi of the proteasomal subunit RPN‐6.1 and saw a significant increase in the biological age of all three samples (Figure 4g). Notably, the effect of BTZ shows a dose dependency. *rpn‐6.1* RNAi has been shown to strongly reduce the lifespan of WT worms (Vilchez et al., 2012), and BTZ supposedly mimics the effects by directly blocking the proteasome and has been shown to dramatically reduce the lifespan of starved worms (Webster et al., 2017). Moreover, although no direct lifespan data are available for normal‐fed worms, 10 µM BTZ leads to an early death starting from day 3 (Finger et al., 2019), while 25 µM even increased mortality (Fabian Finger, personal communication). Next, we tested samples with a mutation in *alh‐6* (Yen et al., 2020), which resulted in a 35.7% reduction in the predicted lifespan (Figure 4h). This is remarkably close to the previously reported 33.5% lifespan reduction in *alh‐6(lax105)* (Pang & Curran, 2014). Lastly, we tested *glp‐1* and *nhr‐49*; *glp‐1* samples for which no direct lifespan measurement was available. A mutation in *nhr‐49* was previously reported to decrease the lifespan in a *glp‐1* background by 50–60.69% (Ratnappan et al., 2014), which is in line with the predicted mean 63.96% decrease (Figure 4i).

These results demonstrate that the nested cross‐validation was sufficient to prevent overfitting, that our model extends beyond the data described here and that even lifespan‐affecting stressors unknown to the model, for example, proteasomal stress, are correctly predicted.

We next wondered how well the aging clock that is measured at one specific timepoint could predict the median lifespan. The prediction of the median lifespan from the biological age assumes a uniform lifespan shift. In other words, if the biological age ratio of two strains or treatments stays constant, we are able to compute the predicted median lifespan. For example, if a sample is twice as long lived as its control, we assume a uniform 50% reduction in the biological age compared with the control, regardless of the timepoint of sequencing; that is, the biological age will be half regardless of the chronological age. The aforementioned intrinsic biases in the chronological age and lifespan assays, however, limit the precision of the predicted median lifespan, especially in chronologically younger samples as here the intrinsic experimental error of ±12 h has a greater influence (Figure S8). Nonetheless, the predicted median lifespan is within the theoretical error bounds in most of the tested samples, indicating that not only biological age but also median lifespan could be predicted by the transcriptomic clock (Table S4).

Nonetheless, the aforementioned 41.3% biological age reduction in *daf‐2* in 4‐day adults corresponds to a 1.71‐fold lifespan extension. This *daf‐2* strain is reported to be 2.6‐fold longer‐lived than its control; however, even with the theoretically optimal prediction, the predicted lifespan effect will vary due to the aforementioned intrinsic biases to around 2.6 ±0.5‐fold. Since the WT sample of this dataset (Zarse et al., 2012) was already longer lived than our standard 15.5 days, we also computed the comparison against 15.5 days which resulted in a 2.31‐fold increase in lifespan for *daf‐2*.

In addition, it cannot be excluded *per se* that some mutations or treatments might affect the lifespan non‐uniformly over time, which would result in an additional bias in the model (Table S4). Indeed, our analysis of the 2 DR datasets (Figure 3b and Figure S7a) might indicate such a bias (even though all values are within the lifespan error bounds). The 12.9% reduction in biological age at day 4 (Figure 3b) corresponds to a 1.15‐fold lifespan extension (in comparison with the theoretical 1.36 ± 0.26‐fold extension). The samples on two additional days of DR (Figure S7a), however, are predicted to be 1.47 times longer lived (theoretical 1.61 ± 0.22‐fold extension).

In conclusion, we demonstrated that the BiT age clock of *C. elegans* is highly accurate and versatile usable. We showed that it correctly predicts the effects of insulin‐like signaling, a modified miRNA regulation, the effect of an aberrant active transcription factor, and the reversal of this effect by an epigenetic mark, translational efficiency, dietary restriction, and the requirement of the intact innate immune system on its lifespan‐extending effect, heat stress as well as pathogen exposure, and the effects of diet‐depending metabolites. Lastly, we also showed that the predictor is able to correctly identify the effect of Metformin through the host's microbiota, the dosage‐dependent effect of drugs, and the counterintuitive fact that the combination of lifespan‐extending drugs might not be necessarily synergistic. Strikingly, our model extends beyond the data used for the nested cross‐validation and is able to correctly predict the biological age of worms, for which no direct lifespan data were available. The BiT age clock could thus facilitate the assessment of pro‐ and anti‐aging effects of genetic, metabolic, environmental, or pharmacological interventions as it determines the biological age and predicts median lifespan.

### 2.3 The predictor genes are enriched in age‐related processes, the innate immune response, and neuronal signaling

**FIGURE 5:**
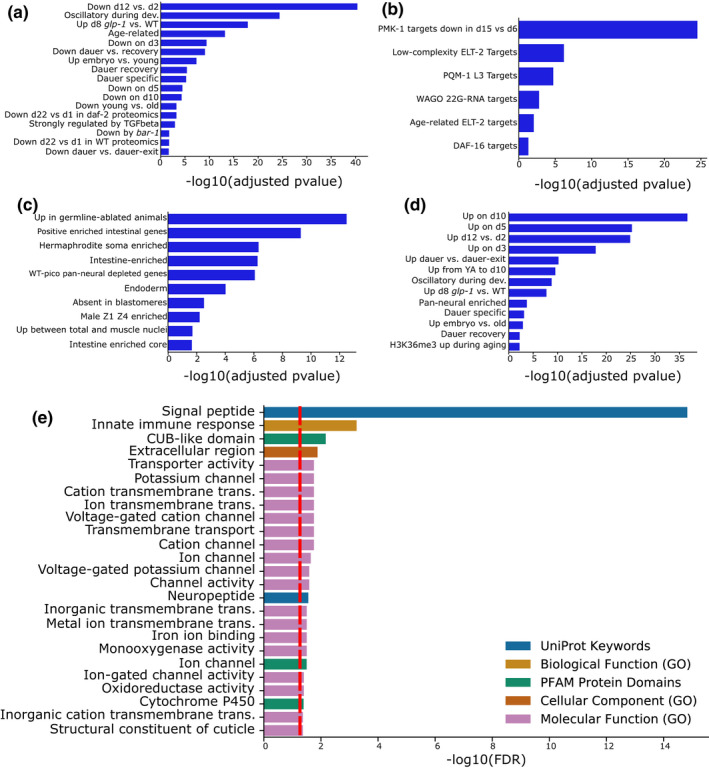
Functional analysis of the predictor genes. (a–d) WormExp gene set enrichment analysis for the 576 predictor genes. The *x*‐axis displays the −log10 of the adjusted *p*‐value. Only statistically significant (adjusted *p* < 0.05) enrichments are shown. (a–c) Gene set enrichment analyses for the genes with a coefficient ≤0 for the Development/Dauer/Aging category (a), the TF Targets category (b), and the Tissue category (c). (d) Gene set enrichment analyses for the genes with a coefficient >0 for the Development/Dauer/Aging category. (e) Functional enrichment analysis for the 576 predictor genes by String and geneSCF. The *x*‐axis displays the −log10 of the FDR. The red line displays an FDR of 0.05. Different enrichment categories are color‐coded

For the final model, we calculated the regression coefficients of the 576 genes based on all the 900 training samples for which lifespan data were available (Figure 1, Table S1). The final regression model utilizes 576 genes, out of which 294 have a negative coefficient and thereby are mostly expressed in young worms, while 282 genes have a positive coefficient and thereby increase the predicted age if active (the genes with the corresponding regression coefficients can be found in Table S2). Intriguingly, the protein‐coding genes with a negative coefficient were enriched on the X‐chromosome and are significantly less expressed from chromosomes I and II (Figure S9a). Protein‐coding genes with a positive coefficient show a opposite trend and are significantly enriched on chromosomes I and II, while depleted from chromosome IV (Figure S9b,c). Interestingly, a gene set enrichment analysis of the genes with a negative coefficient, so those that are associated with younger samples, is enriched in age‐related categories that are downregulated with aging (Figure 5a). Moreover, the 294 genes are enriched in the *pmk*‐*1*, *elt*‐*2*, *pqm*‐*1*, and *daf*‐*16* transcription factor target category (Figure 5b). A motif search at the promoter regions of the genes with a negative coefficient corroborates this finding and shows a significant enrichment in the GATA transcription factors PQM‐1 and ELT‐3 (Figure S10a). Although the gene set enrichment analysis with WormExp did not show a significant enrichment of transcription factors in the gene set with a positive coefficient, the motif search also identified the GATA motif enriched at the promoter regions (Figure S10b). Notably, the GATA transcription factor *elt*‐*6* is within the top 30% of genes with a positive coefficient in our gene set and thereby correlated with older worms and has been shown to increase during normal aging and to increase the lifespan upon knock down by RNAi (Budovskaya et al., 2008). Interestingly, genes associated with younger worms are also enriched in genes that are upregulated in germline‐ablated animals (Figure 5c), which in general exhibit an increased lifespan. Genes with a positive coefficient on the other hand are enriched in categories that show an increase with age (Figure 5d).

A subsequent functional enrichment analysis (s. methods) revealed a strong enrichment of signal peptides (i.e. proteins that are targeted to the secretory pathway by their signal sequence), transporter activity, and neuropeptides, which suggest that especially systemic responses influence the aging process (Figure 5e). Neurotransmitters, although not directly enriched in the GO‐term analysis, might as well play an important role: *hic*‐*1* is one of the genes with the strongest increase in predicted age of our gene set. It has been previously shown to be present at the presynaptic terminal of cholinergic neurons and to regulate the normal secretion of acetylcholine neurotransmitter and Wnt vesicles (Tikiyani et al., 2018). In the same manner, the dopamine receptor *dop*‐*4* is in the top 25% of genes with a negative coefficient and has been shown to promote healthy proteostasis and the innate immunity as well as detoxification genes (Joshi et al., 2016). Interestingly, the innate immune response and cytochrome P450 enrichment in our gene set might indicate a role of a general stress response, detoxification, and drug metabolism during the aging process. Consistent with a general stress response, we also find *csa*‐*1* in the list of genes with a positive coefficient, which might indicate an increased DNA damage load in older worms.

To conclude, these results further validate the genes used for the age prediction and indicate that the aging process might be driven by the dysregulation of single transcription factors (Figure 5b) and a systemic signal transmitted by secreted peptides (Figure 5e).

### 2.4 Improved Human age prediction by the BiT age clock

To demonstrate that our novel approach is also usable for other organisms, we employed a recent human dermal fibroblast RNA‐seq dataset generated from cell culture of 133 healthy individuals with ages between 1 and 94, and 10 patients with Hutchinson‐Gilford progeria syndrome (HGPS) with ages between 2 and 9 (Fleischer et al., 2018). Fleischer et al. showed that an LDA ensemble approach can predict the age of the 133 healthy patients with a *r* ^2^ of 0.81, a mean error of 7.7 years, and a median error of 4.0 years. Moreover, they find a statistical increase in the predicted biological age of HGPS patients, as would be expected from a premature aging disease. However, as they mention, the ensemble method has some limitations, that is, the discretization of age, the computational cost, and the difficult interpretation of the influence of gene expression changes on the predicted age.

**FIGURE 6:**
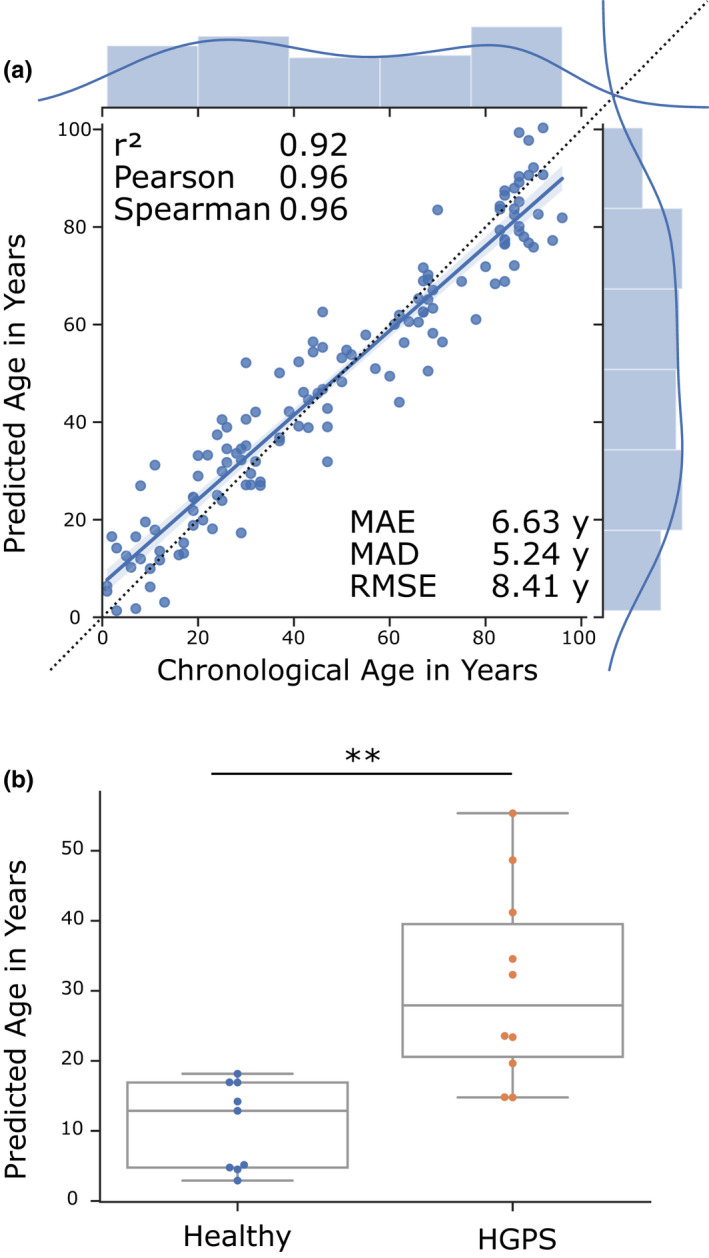
Transcriptomic human aging clock. (a) Results of the age prediction computed by cross‐validation on human fibroblast gene expression data. The *x*‐axis shows the chronological age in years. The *y*‐axis shows the predicted age computed by an elastic net regression on binarized gene expression data. Every blue dot displays one RNA‐seq sample. The regression line with the 95% confidence interval is shown in blue, and the dotted line shows the perfect linear correlation. The distribution of the data is shown on the side of the plot. *r* ^2^ = coefficient of determination, Pearson = Pearson correlation, Spearman = Spearman correlation, MAE = mean absolute error in years, MAD = median absolute deviation in years, RMSE = root‐mean‐square‐error in years. Data from GSE113957. (b) Box plots of age predictions of samples from Hutchinson‐Gilford progeria syndrome patients (red) and predictions of age‐matched healthy controls (blue) by the elastic net regression of binarized gene expression data. Progeria samples are predicted to be significantly older than age‐matched healthy controls. Data from GSE113957. **p ≤ 0.01, calculated by an independent two‐sided *t* test. Table S3 contains more detailed statistics

Our regression‐based method is fast to compute, does not require the discretization of age, and directly allows the effect interpretation of the activity of single genes on the predicted age. Using the elastic net regression on the unbinarized data resulted in a model of 132 predictor genes and in a similar prediction quality as the elastic net regression by Fleischer et al. (Figure S11a), and similarly, the HGPS samples are not predicted to be biologically older (Figure S11b). However, binarization of the data before calculating the elastic net regression improved the results dramatically to an *r* ^2^ of 0.92, a Pearson correlation of 0.96 (*p* = 7.87e‐73), a Spearman correlation of 0.96 (*p* = 9.31e‐73), a MAE of 6.63 years, a MAD of 5.24 years, and a RMSE of 8.41 years (Figure 6a). Moreover, our model predicts the HGPS patients to be significantly older (Figure 6b). This new model contains 141 predictor genes (Table S5), out of which 25 are significantly enriched in the biological process regulation of cell death. Interestingly, among the predictor genes the forkhead transcription factor FOXO1—a regulator of the aging process in *C. elegans* and mammals—is positively correlated with age thus further supporting the evolutionary conservation of transcriptionally regulated longevity mechanisms (Martins et al., 2016).

To summarize, these data indicate that elastic net regression on binarized gene expression data is not only usable in the nematode *C. elegans*, but also in more complex organisms like humans.

## 3 DISCUSSION

The molecular understanding of aging on the genetic, epigenetic, transcriptomic, proteomic, and metabolomic level has made steady progress over the recent years. Since the initial discovery of genetic mechanisms that determine longevity, *C. elegans* has remained an important model system not only for the genetics of aging but also for devising molecular intervention strategies. However, up to date no single model could predict the biological age of any organism to a high accuracy in diverse strains, treatments, and conditions. In our study, we show that the binarization of gene expression data allows a biological age prediction of *C. elegans* to an unprecedented accuracy and for the first time the prediction of a variety of lifespan‐affecting factors. Additionally, we show that the binarization approach, even without the biological rescaling, might be applicable to and improving the predictions in other organisms. This is in contrast to the currently most widely used epigenetic clocks, which are limited to organisms with DNA methylation marks. Moreover, our results suggest that especially the innate immune system and neuronal signaling are important for an accurate prediction and therefore also might play an essential role in the aging process.

Binarization of the gene expression data hugely improved the predictability of the biological age. Interestingly, the biggest deviation from the true biological age is in the samples treated with heat shock or in *mir*‐*71*, *eat*‐*2*, and *skn*‐*1 (gof)* mutants. Heat‐shock treatment and an *eat*‐*2* mutation have been shown to exhibit a different aging trajectory and to diverge from the temporal scaling approach proposed by Stroustrup (Stroustrup et al., 2016). Similarly, *skn*‐*1 (gof)* and *mir*‐*71* display a sharp drop in lifespan (Inukai et al., 2018; Nhan et al., 2019) that cannot totally be accounted for with our median lifespan‐rescaling approach. Incorporating the whole lifespan curve could therefore improve the prediction even further. In this regard, it is also noteworthy that the utilized bulk‐sequencing data introduce several biases that might not be reflected in a simple rescaling approach. We tried to alleviate some of the potential biases with our second rescaling approach, which should reduce the error that is introduced by the fact that especially the biologically older part of a population dies off first. However, it has been published that *C. elegans* dies of at least two different types of death (Zhao et al., 2017): either an early death with a swollen pharynx, induced by an increased bacterial content, or a later death with an atrophied pharynx. This might introduce a different bias, since the initial transcriptional response close to an early death might be different from the response to a later death. Nevertheless, even with these limitations our model predicts the biological age of worms remarkably well.

The increasing error and increase in variance of the age predictor in older worms is especially visible in the unbinarized model. This might be due to the known age‐dependent increase in transcriptional variety that limits the ability of the regression model to pick an accurate subset of genes. Different hypotheses have been proposed that try to explain this transcriptional noise. In *C. elegans*, it might be partially regulated by a microRNA feedback loop that is dependent on *mir*‐*71* (Inukai et al., 2018), serotonergic signals (Rangaraju et al., 2015), and the decline of the GATA transcription factor ELT‐2 during aging (Mann et al., 2016). One interesting possibility is the idea that the increasing noise is driven by accumulating somatic mutations over the course of aging. Indeed, Enge et al. demonstrated an increase in the transcriptional noise as well as an age‐dependent accumulation of somatic mutations in single human pancreatic cells; however, they did not find any support for a causal relationship between exonic mutations and transcriptional dysregulation (Enge et al., 2017).

### 3.1 Transcription factors

Similar to Tarkhov et al., we find an enrichment in targets of DAF‐16, the GATA transcription factors PQM‐1 and ELT‐2, and PMK‐1 in our predictor gene set. DAF‐16 is known to be involved in a variety of stress responses and longevity pathways (Sun et al., 2017). GATA transcription factors have been found to be relevant for a variety of tissue‐specific stress responses and to have a functional role in the aging process (Budovskaya et al., 2008). Moreover, deactivation of *elt*‐*2* has been described as a major driver of normal *C. elegans* aging (Mann et al., 2016) and *pqm*‐*1* has been shown to decline with age and to be involved in *daf*‐*2*‐mediated longevity (Tepper et al., 2013). The p38 MAPK family member *pmk*‐*1* is an important gene in the nematode's pathogen defense system and innate immunity.

### 3.2 Innate immune response

The innate immune system of *C. elegans* has been linked to several lifespan‐affecting pathways (Ermolaeva & Schumacher, 2014). Schmeisser et al. (2013), for example, showed that dietary restriction (DR)‐dependent lifespan extension requires a limited neuronal ROS signaling via a reduced mitochondrial complex 1 activity that activates PMK‐1/p38. Furthermore, it has been shown that the intestinally produced and secreted innate immunity‐related protein IRG‐7 can lead to the activation of the p38‐ATF‐7 pathway and is required for the longevity in germlineless nematodes (Yunger et al., 2017). Apart from long‐lived mutants, PMK‐1 expression was also observed to decline with normal age, leading to an innate immunosenescence in *C. elegans* that has been proposed to be a driving factor of the aging process (Youngman et al., 2011). This immunosenescence and the overall involvement of the innate immune system in aging has also been shown in other model organisms and might demonstrate an evolutionary conservation. Our work falls in line with these reports and supports an important role of the innate immune response in *C. elegans* aging.

### 3.3 Neuronal signaling

Our model also shows an enrichment in neuropeptide signaling. Neuronal communication is important for the organism's homeostasis when responding to different stressors and a changing environment and has been implicated in the aging process. It has also recently been shown that the suppression of excitatory neurotransmitter and neuropeptide signaling is partially required for the longevity of *daf*‐*2* mutants (Zullo et al., 2019) and similarly a glia‐derived neuropeptide signaling pathway that affects the aging rate and healthspan of worms has been described and shows the potential for neuropeptide involvement in the aging process (Yin et al., 2017). In line with this, we find *hic*‐*1* and *dop*‐*4* in our predictor gene set. *hic*‐*1* is important for the regulation of acetylcholine neurotransmitter (Tikiyani et al., 2018) and might therefore indicate a role of *hic*‐*1* in the locomotion defect that occurs with aging (Glenn et al., 2004). Besides the role of *dop*‐*4* in the innate immune response (Joshi et al., 2016), it has also been implicated in the slowing down of habituation (Ardiel et al., 2016). Older worms have been shown to exhibit a greater habituation and a slower recovery from it (Beck & Rankin, 1993). The fact that *dop*‐*4* has a negative coefficient in our age prediction suggests that it is less transcribed in older worm populations, thereby making it an interesting target for the cause of increasing habituation with age.

### 3.4 Human data

Lastly, we demonstrated that binarized gene expression data also allow building an accurate human age prediction. Currently, the analysis is limited by the data amount and future studies should include more high‐quality data from different cohorts with different environments and populations. Optimally, the data would be generated with biopsies from different tissues of living donors without the need of cell culture. Nevertheless, we demonstrated that binarization improves the level of prediction beyond the current standard and that it also allows for a prediction by an elastic net regression, which results in an easy interpretable gene set. Interestingly, we found a significant enrichment in the biological process regulation of cell death, including FOXO1, which indicates that certain age‐related pathways, such as insulin signaling, are indeed relevant for multiple species and evolutionarily conserved.

## 4 CONCLUSIONS

The binarized expression of our 576 genes is sufficient to predict the biological age of *C. elegans* independent of the underlying genetics or environment with an accuracy near the theoretical limit. Our analysis suggests that the innate immune response, neuronal signaling, and single transcription factors are major regulators of the aging process independent of the strain and treatment. Although the temporal rescaling approaches will not be applicable in humans, we have also shown how the binarization approach improves the chronological age prediction of a recent human dataset. Our work establishes that an accurate aging predictor can be built on binarized transcriptomic data that extends beyond the training data, predicts lifespan effects across diverse genetic, environmental, or therapeutic interventions, is employable in distinct species, and might thus serve as a universally applicable aging clock.

## 5 MATERIALS AND METHODS

### 5.1 Data processing

The quality of the data was checked with FastQC, and the data were preprocessed with Fastp with the following parameters: ‐g to trim polyG read tails caused by sequencing artifacts, ‐x to trim polyX, ‐q 30 for base quality filtering, and ‐e 30 to filter for an average quality score. Paired‐end samples were processed together. After preprocessing, the samples were mapped with STAR‐2.7.1a with the following parameters: ‐‐outFilterType BySJout ‐‐outFilterMultimapNmax 20 ‐‐alignSJoverhangMin 8 ‐‐alignSJDBoverhangMin 1 ‐‐outFilterMismatchNmax 999 ‐‐outFilterMismatchNoverReadLmax 0.04 ‐‐alignIntronMin 20 ‐‐alignIntronMax 1000000 ‐‐alignMatesGapMax 1000000 ‐‐quantMode GeneCounts.

The genome directories were generated with the ce11 genome, WBcel235.96 without rRNA and the parameter –genomeSAindexNbases 12 for *C. elegans* and the hg38 genome, GRCh38.97 without rRNA, and the parameter –genomeSAindexNbases 14 for human data. The parameter –sjdbOverhang was set to the read length of the sample −1.

The validation samples with the IDs GSE106079, GSE127917, GSE138129, and GSE141041 were mapped with Salmon‐1.1 with a k‐mer length of 31 and the following parameters: ‐l A –validateMappings –gcBias –seqBias.

The raw counts for the validation samples with the IDs GSE93826 and GSE138035 were directly downloaded from the gene expression omnibus.

The counts for unstranded RNA‐seq were merged into one csv file, and edgeR was used to generate count per millions (CPM).

Functional enrichment analysis was done with String v.11 and geneSCF, and the gene set enrichment analysis with WormExp.

### 5.2 Binarization

To binarize the data first zero CPMs were masked by NaN. For the remaining data, the median for each sample was calculated and genes bigger the median were set to 1, while genes smaller or equal to the median were set to 0, finally genes masked by NaN were set to 0 as well.

### 5.3 Temporal rescaling

For the temporal rescaling, we set the median lifespan of a standard worm to 15.5 days of adulthood. We calculated a correction factor for every sample by dividing this standard lifespan by the median lifespan reported by the publication of the corresponding sample. We restricted the training data to this subset of samples for which a lifespan was reported in the associated publication, because even a wild‐type worm under standard conditions can show dramatically different median lifespans in between different laboratories. For example, the median lifespan of N2 wild‐type worms at the same standard conditions in the datasets we used ranges from 15 days in GSE112753 to 24 days in PRJNA508378, which increases to a range from 14 days (GSE65765) to 30.55 days (GSE92902) just by including FUDR‐treated worms. Without requiring the lifespan data from the same publication and just setting the lifespan to the standard 15.5 days, we would introduce a twofold bias in the rescaled biological age, which would reduce the prediction of the model accordingly. The chronological age of each sample is multiplied with this correction factor to result in the approximated biological age of the sample. The chronological age, correction factor, and biological age for every sample can be seen in Table S1.

The datasets GSE106079 and GSE93826 were not associated with any publication and thereby no lifespan data were available. However, both datasets consist of a time course of *C. elegans* aging and would therefore be valuable validation data. Since the strains used in both datasets should not show strong deviations in the median lifespan from wild‐type worms, we assumed that the lifespan is 15.5 days in both cases. Since this lifespan is approximated and should therefore include a bias as shown above, we would expect the prediction error to be higher than usual.

### 5.4 2nd rescaling approach

For the 2nd rescaling of the biological age, we set the maximum biological age of the worm to 15.5 days. Assuming a normal distribution of biological age around the chronological age of a worm population and further assuming that, on average, worms will die according to their biological age, we can assume that the maximum biological age of a worm is the median lifespan of 15.5 days. Worms living longer than the median lifespan were biologically younger and therefore did not cross the line of 15.5 days (see Figure S3). Since the first wild‐type worms under standard conditions start dying at around 9 days of adulthood, the oldest worms at day 8 should be biologically around 15.5 days old. Therefore, we approximated the standard deviation to be 8/3. Centering a normal distribution at 8 days with a SD of 8/3 will contain 99.73% of the area under the curve within day 0 to day 16.

Next, we approximated that the biological age distribution is not changing over time and that the SD over 8/3 stays stable. To calculate the median of the data after trimming the data at the maximum age of 15.5 days, we first need to calculate how much data are trimmed. We approximate this by utilizing the error function:

$$\text{erf}\left(z\right)=\frac{2}{\sqrt{\pi }}\int_{0}^{z}e^{‐t^{2}}dt$$

implemented in the SciPy library.

The approximation of the percentage *p* of data that is remaining on the left side from the maximum lifespan of 15.5 days on the biological age x is as follows:

$$p=\frac{1}{2}\text{erf}\left(\frac{\frac{15.5‐x}{\text{8}\;/\text{3}}}{\sqrt{2}}\right)+0.5$$

Here, $\frac{15.5‐x}{8/3}$ calculates how many SDs the biological age is apart from the maximum age of 15.5 days. And $\text{erf}\left(\frac{\frac{15.5‐x}{\text{8}\;/\text{3}}}{\sqrt{2}}\right)$ calculates the percentage of the area under the bell curve for the calculated number of SDs. If the biological age would be one SD away from the maximum age of 15.5 days, that is, 8/3 days, the area under the curve would be ~68.2%. However, this value corresponds to the area on the left and the right of the median. Since we are only interested in one side, we have to divide the area by 2 and add 50%, that is, 0.5, for the opposite side. With this, *p* will approximate the area under the curve that is remaining after trimming the right side from the maximum lifespan of 15.5 days.

To get the approximation of the new median percentage for the trimmed bell curve, we can divide *p* by 2. This new median percentage can be used to calculate the median in days by reverting the calculation. First, we subtract the new median percentage from 0.5 to get the deviation from the original median percentage, that is, 0.5, and use the inverse error function to approximate *s*, the number of standard deviations that the new median is shifted to the left of the old median:

$$s={\text{erf}}^{‐1}\left(0.5‐\frac{p}{2}\right)\sqrt{2}*2$$

The new median *m*, in other words the new rescaled biological age, can then be calculated by the following:

$$m=x‐s*\frac{8}{3}$$

where 8/3 is the standard deviation that we set in the beginning and *x* the biological age, that is, the original median.

### 5.5 Model fitting—Parameter search

The age prediction models use an elastic net regression as implemented by Pythons’ sklearn. The random_state was set to 0, the max_iter to 1,000, and positive=False. The best parameter settings for alpha and the L1/L2 ratio were selected using a parameter grid search with a nested cross‐validation approach. To avoid overfitting during the training, we split the data into multiple partitions. Every sample of the same genetic background, with the same treatment, and RNA interference of the same rounded biological age to days was considered to be one partition. This makes sure that samples with a similar transcriptome are taken out together during the process. The elastic net regression is trained on the remaining data, and the partition that got taken out will be predicted. To get an overview of the accuracy of the model, this process is repeated for the partitions in the dataset. In the end, every sample will be predicted exactly once, which allows the comparison of the predicted with the true biological age.

A simple cross‐validation like this gives an overview of the accuracy of the model; however, to select the best parameter setting, a nested cross‐validation is required, since otherwise information may leak into the model and introduce another type of overfitting. Therefore, after splitting the data into the test and the train partitions (the outer loop), the latter will be split again into an inner test and train partition (the inner loop). This inner cross‐validation will be computed for every parameter set to compute the average of the absolute error for each parameter setting.

This will be done for every partitioning in the outer loop to select the most stable parameter set. The parameters selected by this approach for the binarized data are alpha = 0.075 and l1_ratio = 0.3.

### 5.6 Model fitting—Optimal gene set

To obtain the optimal gene set without overfitting, a similar approach was taken. Instead of looping over different parameter settings, the cross‐validation for the gene set loops over a list of the genes with the highest absolute coefficients. First, for every training partition in the outer loop the full model with alpha = 0.075 and l1_ratio = 0.3 is computed. This will result in a model, where every gene is annotated with a coefficient. In the binarized model, the sum of the coefficients for all genes that are 1 in the sample added to the intercept equals the predicted age. Therefore, a negative coefficient will result in a younger predicted age, while a positive coefficient will increase the predicted age. Next, we loop over different subsets of the top genes to identify the approximately optimal and smallest gene set for the given partition. For every gene set, the inner cross‐validation loop is computed and the gene set with the smallest average absolute error is saved. This will be done again for every partition in the outer loop to gain multiple gene sets. Similar to the parameter search, the most stable gene set is taken by retaining only those genes that were used by every partition. This stable gene set selected by this approach for the binarized data after the second rescaling are the 576 genes described in Table S2. This final model starts at an intercept of 103.55 hrs (4.31 days).

### 5.7 Using the clock

To predict the biological age of new data, one has to start with binarizing the transcriptome as described above. The elastic net coefficients (column 2 in Table S2) are added up for all of the 576 genes with a value of 1 after binarization. Finally, the intercept of 103.55 hr has to be added to get the final prediction of the biological age in hours. The code is included in https://github.com/Meyer‐DH/AgingClock/

### 5.8 Motif search

The set of genes with a coefficient >0, respective ≤0, was used as input for the findMotifs function of Homer‐4.9.1–6 with the parameters ‐len 8,10 ‐start −300 ‐end 100. To make sure that the maximum number of genes got recognized by Homer, we first converted the Wormbase IDs to the sequence name with WormBase's SimpleMine and added “CELE_” in front of it. These identifiers were then searched in the “worm.description” file of Homer to gain the corresponding RefSeq IDs that are recognized by the program. The p‐values were calculated with a hypergeometric test.

### 5.9 Median lifespan fold change prediction

The median lifespan fold change can be predicted by the biological age of the strain of interest and its control, assuming a uniform age effect. The median lifespan of each strain can be computed by dividing the chronological age by the biological age and multiplying it by 15.5 days. To compute the fold change, the median lifespan of interest is divided by the control lifespan, or easier, the biological age of the strain of interest can be divided by the biological age of the control, if the chronological age is the same.

The theoretical range of lifespan fold change predictions in Figure S8 was calculated with the Python package Uncertainties. The chronological age bias was set to 0.5 days and the lifespan assay bias to 5%. The code is included in https://github.com/Meyer‐DH/AgingClock/

### 5.10 Figure details

All plots were done with Seaborn‐0.9.0. Boxplots: The center line represents the median; the box limits the bottom, and top quartiles of the data and the whiskers show the 1.5x interquartile range.

### 5.11 Statistics

ANOVA and *t* tests were computed with Python's pingouin library v.0.3.3. post hoc Tukey test were computed with Python's Statsmodels library v.0.10.1.

### 5.12 Citations of the age predictors from the literature

Because currently no general consensus of quality assessment exists and different measurements are being reported, we state the measurements as reported in the cited paper in the introduction. Some of the most common used assessments are as follows:

1. Mean absolute error (MAE): the mean of the absolute difference in predicted and true age.
2. Root‐mean‐square‐error (RMSE): the square root of the average squared differences. Larger errors have a larger effect on the RMSE than on MAE.
3. Median absolute deviation (MAD): the median absolute difference in predicted and true age.
4. Pearson correlation (*r*): measurement of how the predicted and true age changes together. Evaluates linear relationships.
5. Spearman correlation (*r*): similar to Pearson correlation, but evaluates the monotonic relationship. Other than Pearson correlation, the variables do not need to change at a linear rate.
6. Coefficient of determination (*r* ^2^): the fraction of the variance that is predictable with the model. Often the *r* ^2^ is the square of the correlation coefficient; however, this is not true in the general case. The value can get negative if the model fits worse than a horizontal line.

## CONFLICT OF INTEREST

The authors declare no competing interests.

## AUTHOR CONTRIBUTIONS

D.M. conceived and designed the study and performed all bioinformatics analysis; B.S. coordinated the project and together with D.M. designed the study. All authors wrote the paper.

## CODE AVAILABILITY STATEMENT

Code for the binarization, age‐correction, and the prediction of new samples can be found on https://github.com/Meyer‐DH/AgingClock/.

## Supporting information

FigS1‐S11

Supplementary Material

Supporting Information Legends

Table S1

Table S2

Table S3

Table S4

Table S5

## Data Availability

We used publicly available datasets. The details can be found in Table S1.
