# Supplementary material for: BiT age: A transcriptome‐based aging clock near the theoretical limit of accuracy
Source: Aging Cell. 2021 Mar 3;20(3):e13320. doi: 10.1111/acel.13320 (PMC7963339; doi:10.1111/acel.13320)
Supplement: Supplementary file 1 — FigS1‐S11 [file ACEL-20-e13320-s008.pdf]

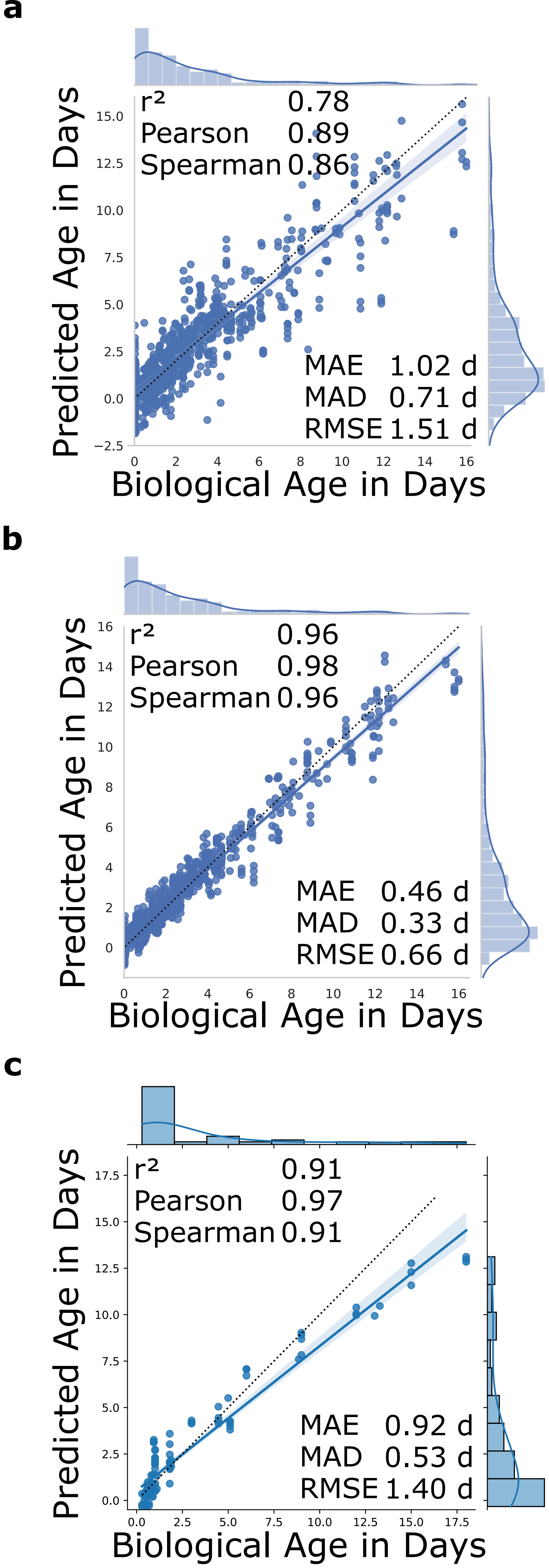

Fig. S1 **Meyer and Schumacher**

a

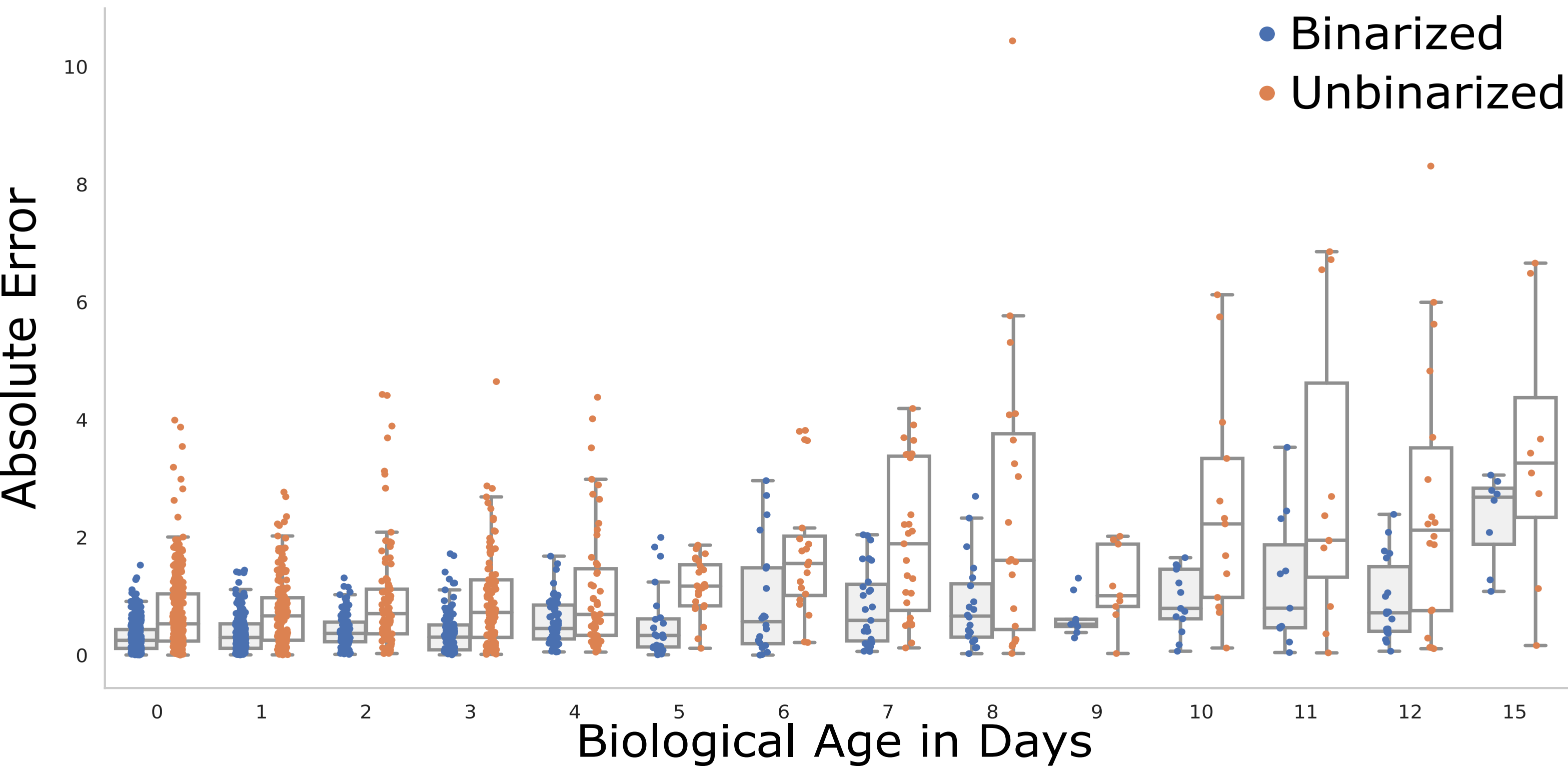

b

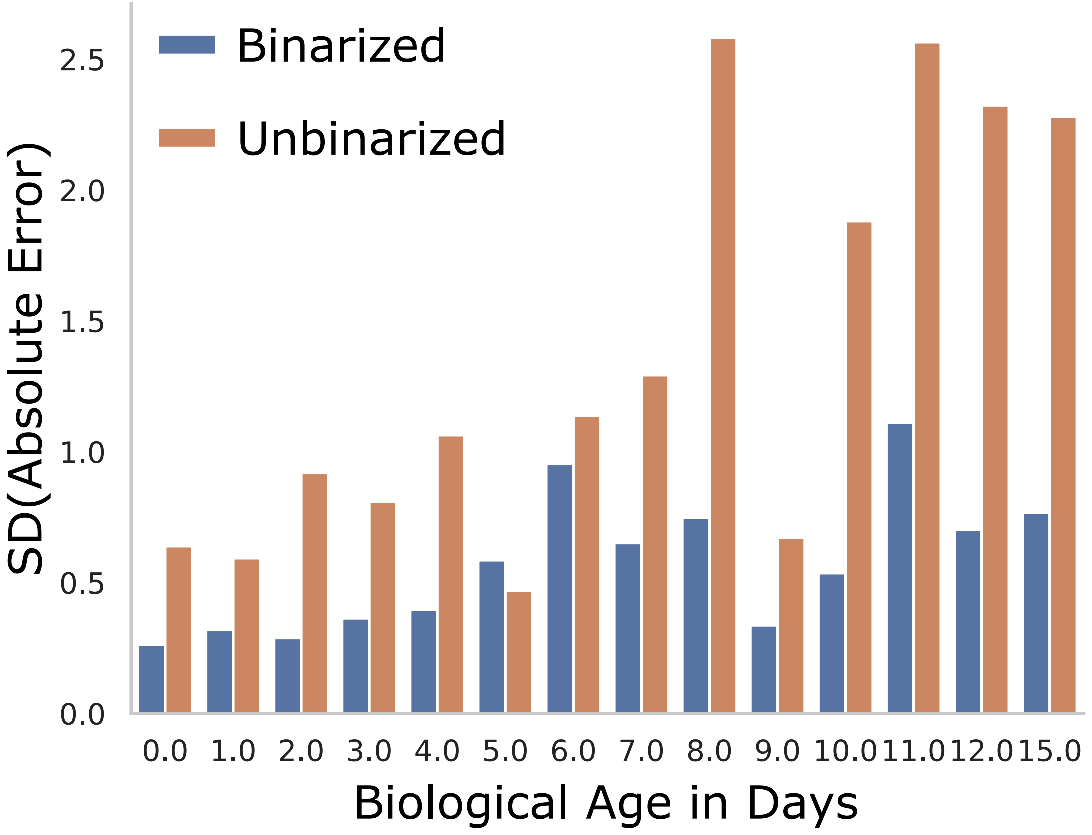

Fig. S2 **Meyer and Schumacher**

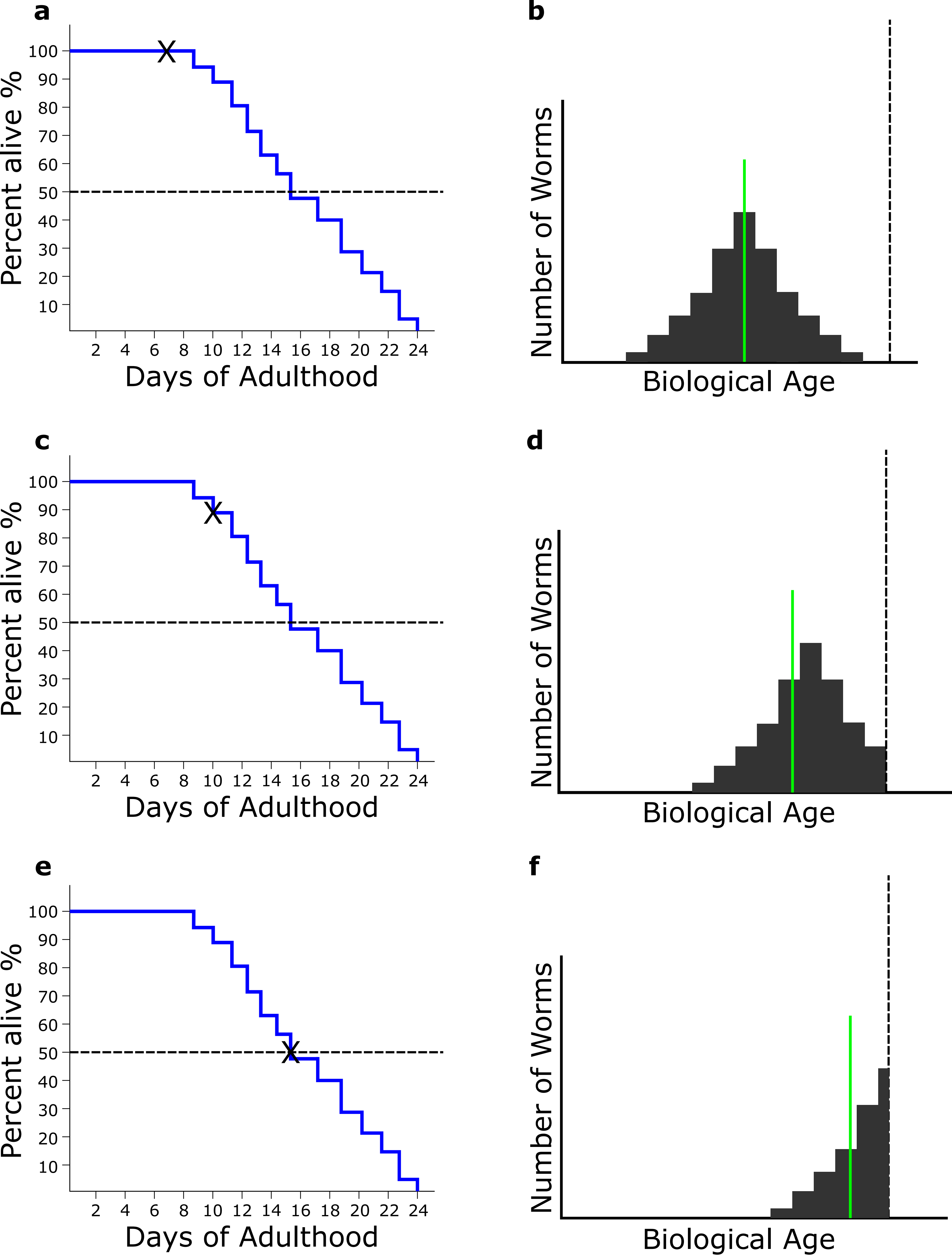

Fig. S3 **Meyer and Schumacher**

a

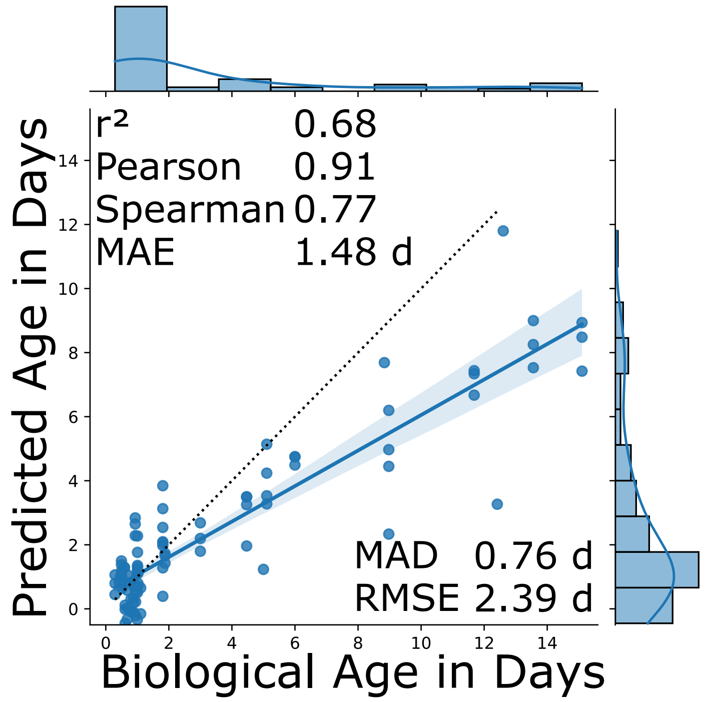

b

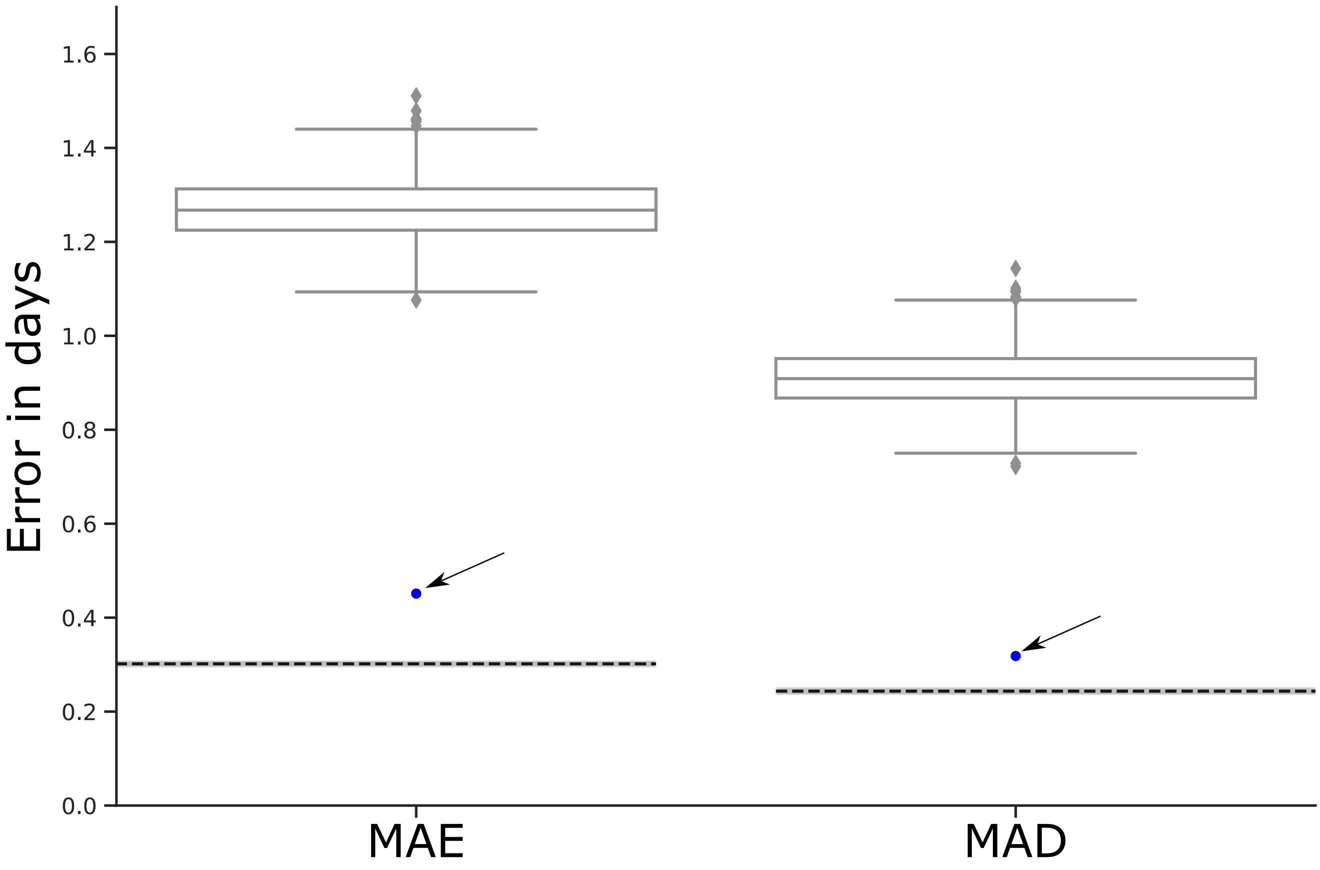

Fig. S4 **Meyer and Schumacher**

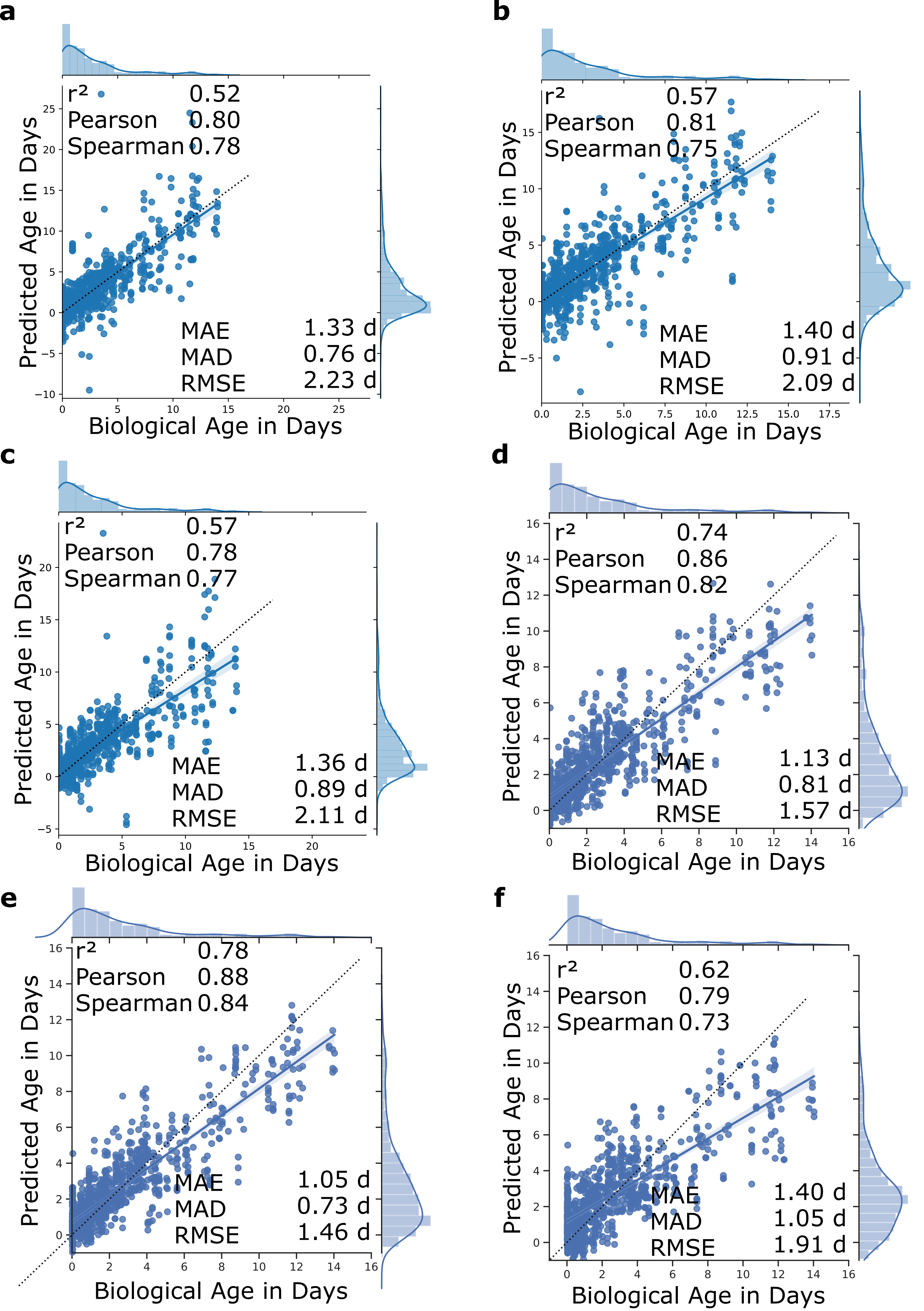

Fig. S5 **Meyer and Schumacher**

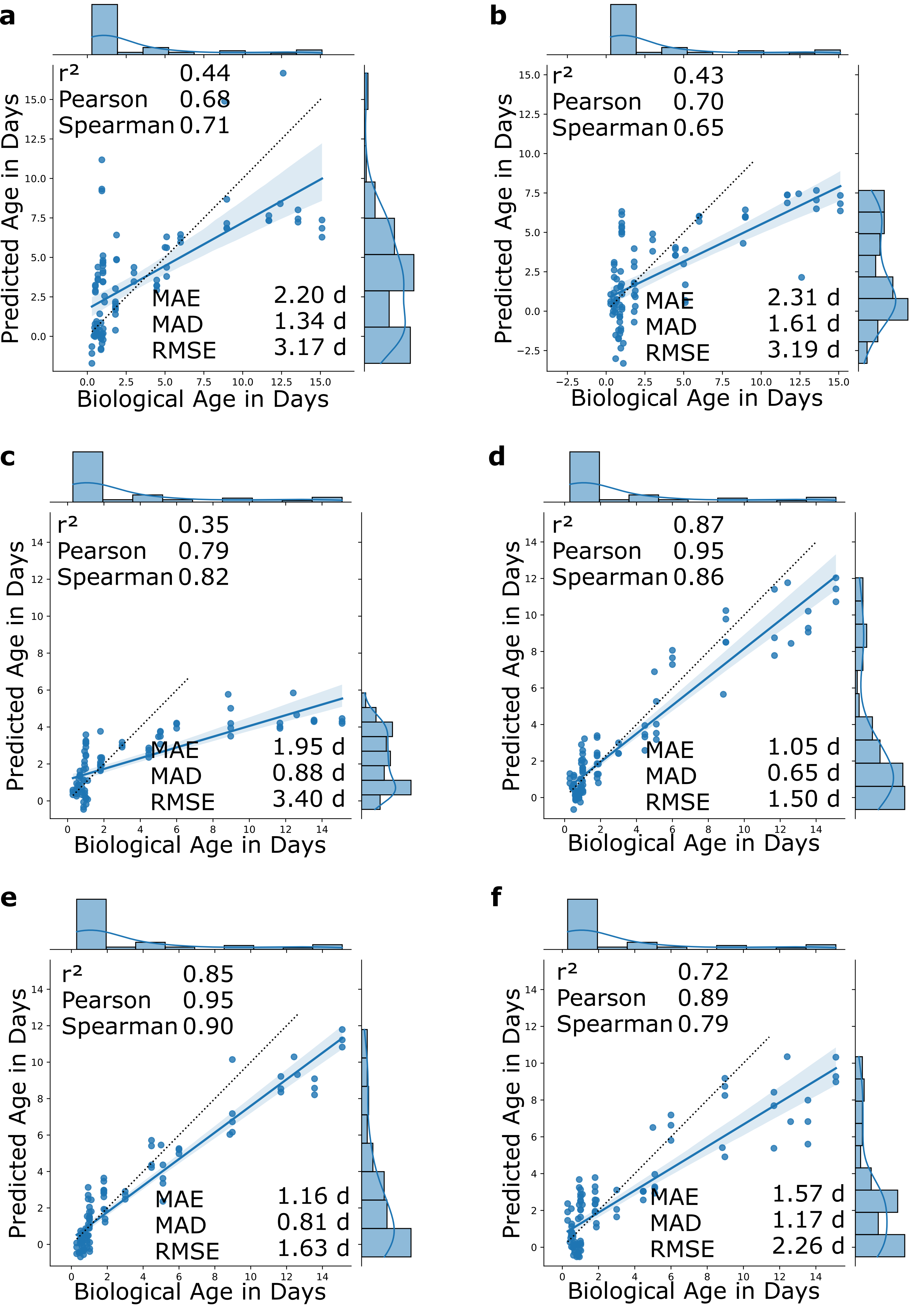

Fig. S6 **Meyer and Schumacher**

a

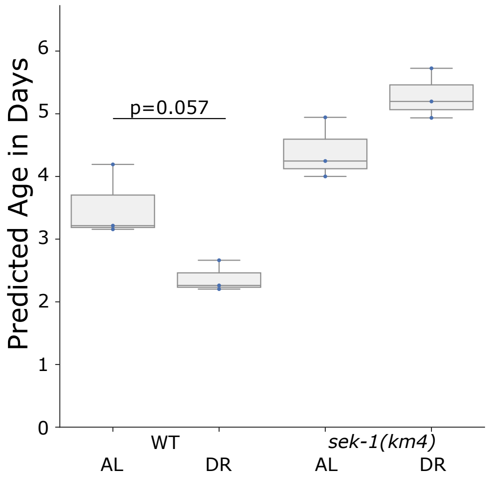

b

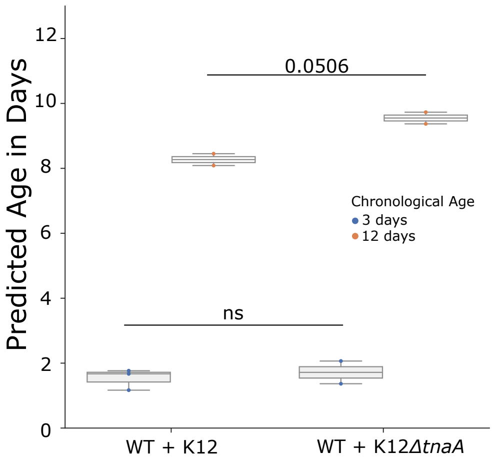

Fig. S7 **Meyer and Schumacher**

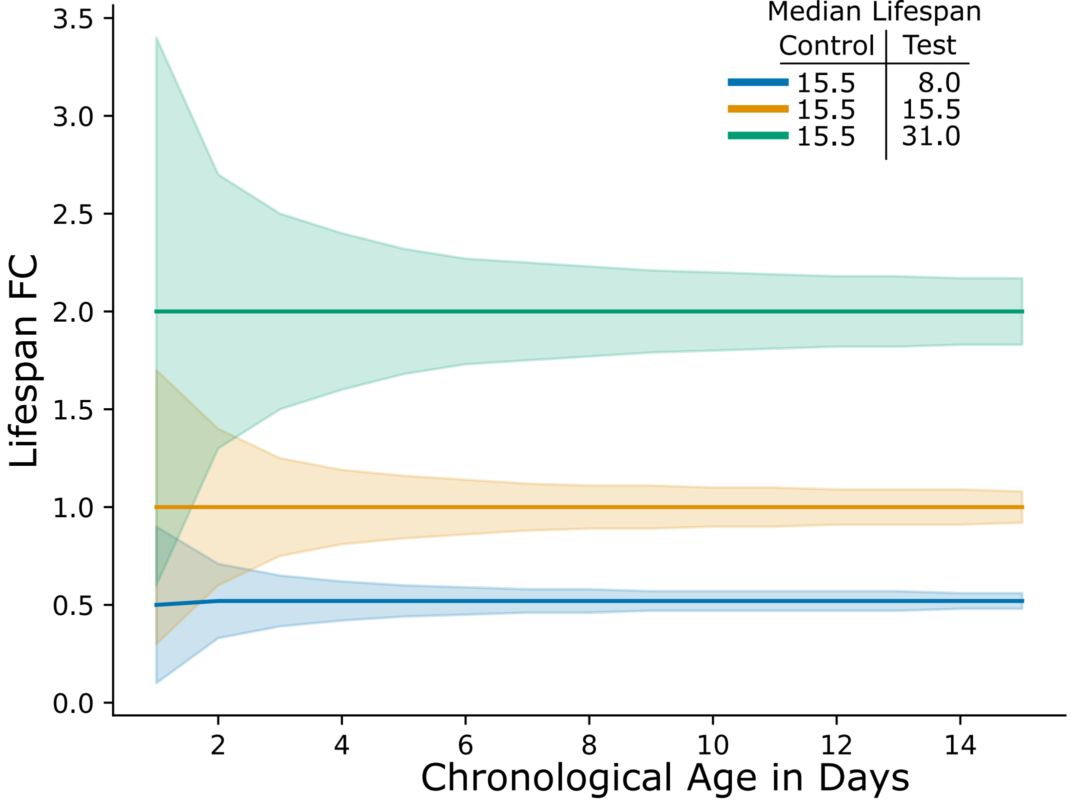

Fig. S8

Meyer and Schumacher

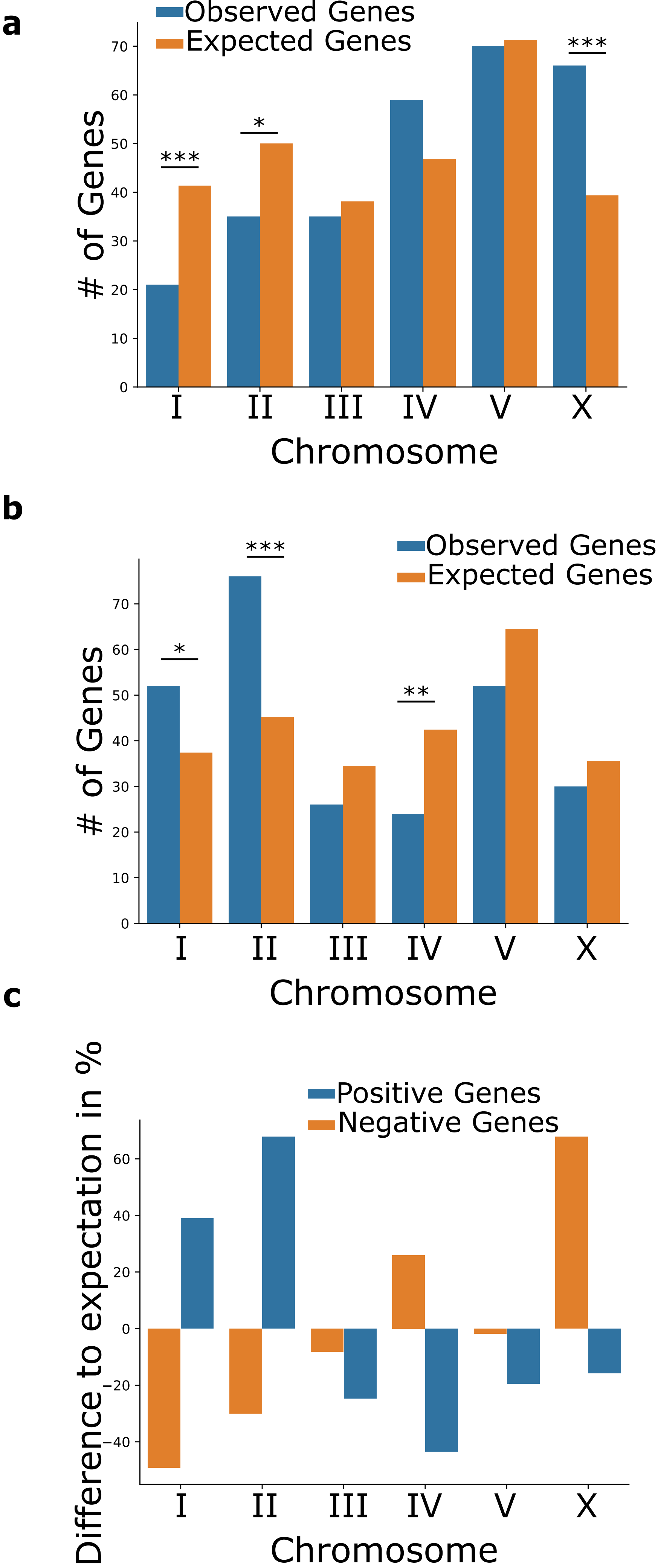

Fig. S9 **Meyer and Schumacher**

a

Name	Motif	% of Genes	% of BG	FC	p-value	q-value
PQM-1(Gata)	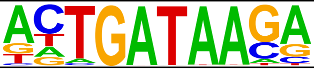	29.93	17.10	1.75	1.09E-07	1.2E-06
ELT-3(Gata)	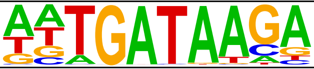	31.02	20.59	1.51	3.00E-05	1.65E-04

b

Name	Motif	% of Genes	% of BG	FC	p-value	q-value
ELT-3(Gata)	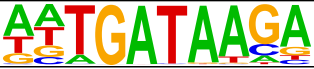	30.04	19.91	1.51	7.74E-05	8.51E-04
PQM-1(Gata)	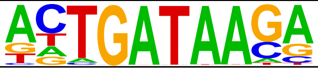	23.72	16.52	1.44	1.97E-03	0.01

a

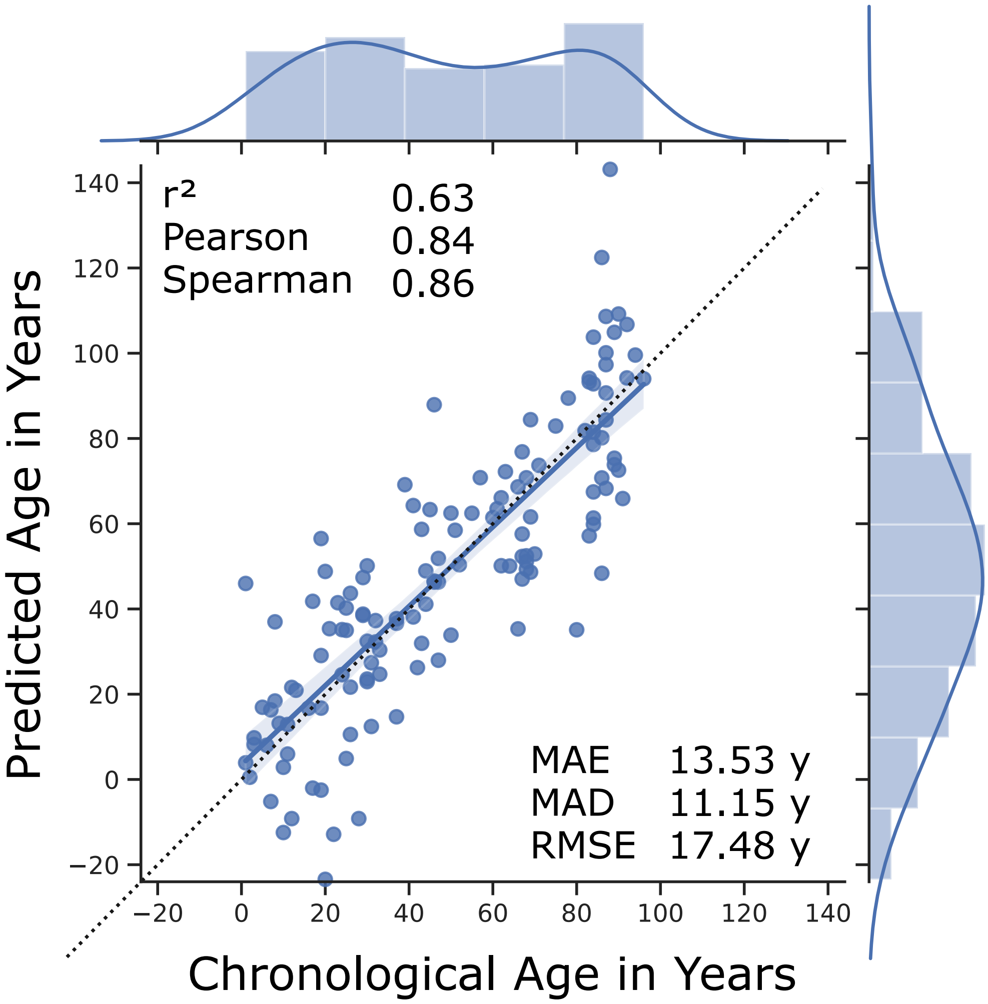
r²
0.63
Pearson
0.84
Spearman
0.86
MAE
13.53 y
MAD
11.15 y
RMSE
17.48 y
Predicted Age in Years
Chronological Age in Years
140
120
100
80
60
40
20
0
−20

b

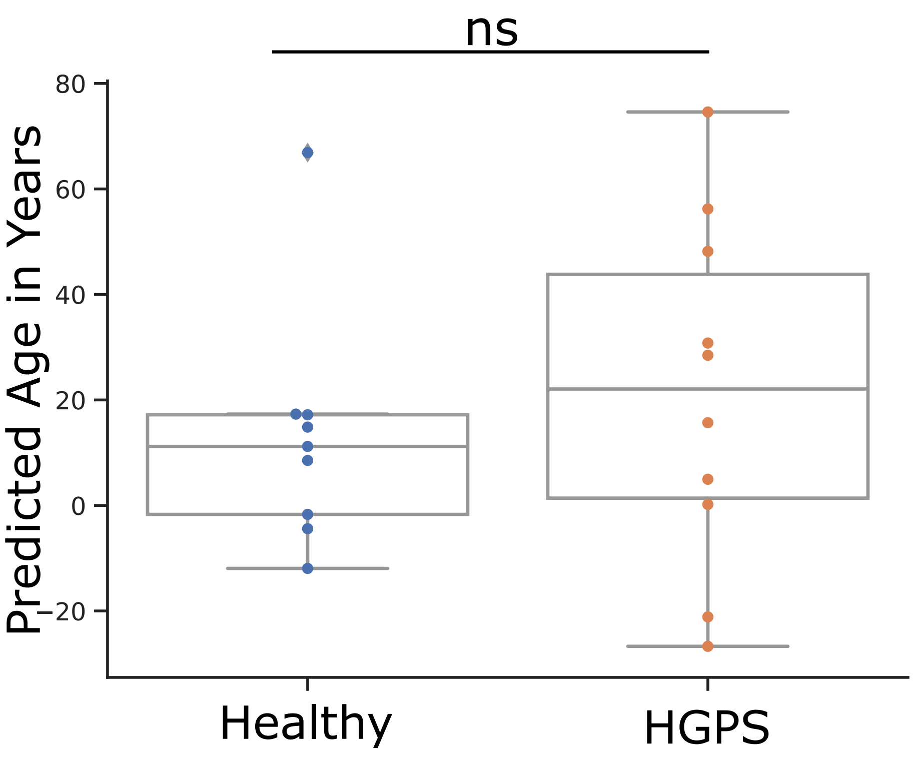
ns
Predicted Age in Years
80
60
40
20
0
−20
Healthy
HGPS
