# Supplementary material for: BiT age: A transcriptome‐based aging clock near the theoretical limit of accuracy
Source: Aging Cell. 2021 Mar 3;20(3):e13320. doi: 10.1111/acel.13320 (PMC7963339; doi:10.1111/acel.13320)
Supplement: Supplementary file 2 — Supplementary Material [file ACEL-20-e13320-s007.docx]

# Supplementary materials and methods

## Programs and methods citations

The following programs and methods have been used in this study:

FastQC (Andrews et al. 2010), Fastp (Chen et al. 2018), STAR-2.7.1a (Dobin et al. 2013), Salmon-1.1 (Patro et al. 2017), edgeR (Robinson et al. 2009), String v.11 (Szklarczyk et al. 2019), geneSCF (Subhash & Kanduri 2016), WormBase’s SimpleMine (Harris et al. 2020), Homer-4.9.1-6 (Heinz et al. 2010), WormExp (Yang et al. 2016).

The following Python libraries have been used:

pingouin v.0.3.3 (Vallat 2018), Statsmodels v.0.10.1 (Seabold & Perktold 2010), Scipy-1.5.1 (Virtanen et al. 2020), sklearn-0.23.1 (Varoquaux et al. 2011), Uncertanties-3.1.1 (Lebigot n.d.), seaborn-0.9.0 (Waskom et al. 2018)

Datasets have been downloaded from the gene expression omnibus (Edgar et al. 2002).

# References

Andrews S, Krueger F, Segonds-Pichon, Anne Biggins L, Krueger C & Wingett S (2010) FastQC. Available at: http://www.bioinformatics.babraham.ac.uk/projects/fastqc.

Chen S, Zhou Y, Chen Y & Gu J (2018) Fastp: An ultra-fast all-in-one FASTQ preprocessor. *Bioinformatics* 34, i884–i890.

Dobin A, Davis CA, Schlesinger F, Drenkow J, Zaleski C, Jha S, Batut P, Chaisson M & Gingeras TR (2013) STAR: Ultrafast universal RNA-seq aligner. *Bioinformatics* 29, 15–21.

Edgar R, Domrachev M & Lash AE (2002) Gene Expression Omnibus: NCBI gene expression and hybridization array data repository. *Nucleic Acids Res.* 30, 207–210. Available at: https://academic.oup.com/nar/article-lookup/doi/10.1093/nar/30.1.207.

Harris TW, Arnaboldi V, Cain S, Chan J, Chen WJ, Cho J, Davis P, Gao S, Grove CA, Kishore R, Lee RYN, Muller HM, Nakamura C, Nuin P, Paulini M, Raciti D, Rodgers FH, Russell M, Schindelman G, Auken K V., Wang Q, Williams G, Wright AJ, Yook K, Howe KL, Schedl T, Stein L & Sternberg PW (2020) WormBase: a modern Model Organism Information Resource. *Nucleic Acids Res.* 48, D762–D767.

Heinz S, Benner C, Spann N, Bertolino E, Lin YC, Laslo P, Cheng JX, Murre C, Singh H & Glass CK (2010) Simple Combinations of Lineage-Determining Transcription Factors Prime cis-Regulatory Elements Required for Macrophage and B Cell Identities. *Mol. Cell* 38, 576–589. Available at: http://dx.doi.org/10.1016/j.molcel.2010.05.004.

Lebigot EO Uncertainties: a Python package for calculations with uncertainties. Available at: https://pythonhosted.org/uncertainties/.

Patro R, Duggal G, Love MI, Irizarry RA & Kingsford C (2017) Salmon provides fast and bias-aware quantification of transcript expression. *Nat. Methods* 14, 417–419. Available at: http://www.nature.com/articles/nmeth.4197.

Robinson MD, McCarthy DJ & Smyth GK (2009) edgeR: A Bioconductor package for differential expression analysis of digital gene expression data. *Bioinformatics* 26, 139–140.

Seabold S & Perktold J (2010) Statsmodels: Econometric and Statistical Modeling with Python. *PROC. 9th PYTHON Sci. CONF*, 57. Available at: http://statsmodels.sourceforge.net/.

Subhash S & Kanduri C (2016) GeneSCF: A real-time based functional enrichment tool with support for multiple organisms. *BMC Bioinformatics* 17, 1–10. Available at: http://dx.doi.org/10.1186/s12859-016-1250-z.

Szklarczyk D, Gable AL, Lyon D, Junge A, Wyder S, Huerta-Cepas J, Simonovic M, Doncheva NT, Morris JH, Bork P, Jensen LJ & Von Mering C (2019) STRING v11: Protein-protein association networks with increased coverage, supporting functional discovery in genome-wide experimental datasets. *Nucleic Acids Res.* 47, D607–D613.

Vallat R (2018) Pingouin: statistics in Python. *J. Open Source Softw.* 3, 1026.

Varoquaux G, Buitinck L, Louppe G, Grisel O, Pedregosa F & Mueller A (2011) Scikit-learn: Machine Learning in Python Fabian. *J. Mach. Learn. Res.*

Virtanen P, Gommers R, Oliphant TE, Haberland M, Reddy T, Cournapeau D, Burovski E, Peterson P, Weckesser W, Bright J, van der Walt SJ, Brett M, Wilson J, Millman KJ, Mayorov N, Nelson ARJ, Jones E, Kern R, Larson E, Carey CJ, Polat İ, Feng Y, Moore EW, VanderPlas J, Laxalde D, Perktold J, Cimrman R, Henriksen I, Quintero EA, Harris CR, Archibald AM, Ribeiro AH, Pedregosa F, van Mulbregt P, Vijaykumar A, Bardelli A Pietro, Rothberg A, Hilboll A, Kloeckner A, Scopatz A, Lee A, Rokem A, Woods CN, Fulton C, Masson C, Häggström C, Fitzgerald C, Nicholson DA, Hagen DR, Pasechnik D V., Olivetti E, Martin E, Wieser E, Silva F, Lenders F, Wilhelm F, Young G, Price GA, Ingold GL, Allen GE, Lee GR, Audren H, Probst I, Dietrich JP, Silterra J, Webber JT, Slavič J, Nothman J, Buchner J, Kulick J, Schönberger JL, de Miranda Cardoso JV, Reimer J, Harrington J, Rodríguez JLC, Nunez-Iglesias J, Kuczynski J, Tritz K, Thoma M, Newville M, Kümmerer M, Bolingbroke M, Tartre M, Pak M, Smith NJ, Nowaczyk N, Shebanov N, Pavlyk O, Brodtkorb PA, Lee P, McGibbon RT, Feldbauer R, Lewis S, Tygier S, Sievert S, Vigna S, Peterson S, More S, Pudlik T, Oshima T, Pingel TJ, Robitaille TP, Spura T, Jones TR, Cera T, Leslie T, Zito T, Krauss T, Upadhyay U, Halchenko YO & Vázquez-Baeza Y (2020) SciPy 1.0: fundamental algorithms for scientific computing in Python. *Nat. Methods* 17, 261–272.

Waskom M, Botvinnik O, O’Kane D, Hobson P, Ostblom J, Lukauskas S, Gemperline DC, Augspurger T, Halchenko Y, Cole JB, Warmenhoven J, de Ruiter J, Pye C, Hoyer S, Vanderplas J, Villalba S, Kunter G, Quintero E, Bachant P, Martin M, Meyer K, Miles A, Ram Y, Brunner T, Yarkoni T, Williams ML, Evans C, Fitzgerald C, Brian & Qalieh A (2018) mwaskom/seaborn: v0.9.0 (July 2018). Available at: https://doi.org/10.5281/zenodo.1313201.

Yang W, Dierking K & Schulenburg H (2016) WormExp: A web-based application for a Caenorhabditis elegans-specific gene expression enrichment analysis. *Bioinformatics* 32, 943–945.
