# Supplementary material for: BiT age: A transcriptome‐based aging clock near the theoretical limit of accuracy
Source: Aging Cell. 2021 Mar 3;20(3):e13320. doi: 10.1111/acel.13320 (PMC7963339; doi:10.1111/acel.13320)
Supplement: Supplementary file 3 — Supporting Information Legends [file ACEL-20-e13320-s002.docx]

## Supporting Information Legends

**Figure S1. Alternative models**

(A) Results of the biological age prediction computed by cross-validation. The x-axis shows the rescaled biological age in days starting from adulthood. The y-axis shows the predicted age computed by an elastic net regression on unbinarized CPMs. Every blue dot displays one RNA-seq sample. The regression line with the 95 % confidence interval is shown in blue and the dotted line shows the perfect linear correlation. The distribution of the data is shown on the side of the plot. r²= coefficient of determination, Pearson= Pearson correlation, Spearman= Spearman correlation, MAE= mean absolute error in days, MAD= median absolute deviation in days, RMSE= root-mean-square-error in days.

(B) Results of the biological age prediction computed by cross-validation. The x-axis shows the rescaled biological age in days starting from adulthood. The y-axis shows the predicted age computed by an elastic net regression on binarized gene expression data. Every blue dot displays one RNA-seq sample. The regression line with the 95 % confidence interval is shown in blue and the dotted line shows the perfect linear correlation. The distribution of the data is shown on the side of the plot. r²= coefficient of determination, Pearson= Pearson correlation, Spearman= Spearman correlation, MAE= mean absolute error in days, MAD= median absolute deviation in days, RMSE= root-mean-square-error in days.

(C) Prediction of the model on 8 independent datasets consisting of 94 samples at different time points. The x-axis shows the biological age in days starting from adulthood before the second rescaling approach. The y-axis shows the predicted age computed by an elastic net regression on binarized gene expression data. For more details on the datasets see the Table S1.

**Figure S2. Comparison of the binarized and unbinarized model error**

(A) The absolute error distribution between the predicted and true biological age is plotted for either the unbinarized (red) or binarized (blue) data. The x-axis shows the true biological age in days. The y-axis the absolute error in days. While the unbinarized model strongly increases the absolute prediction error with age, the increase is less pronounced with the binarized model.

(B) The bar plots show the standard deviation of the absolute prediction errors in days. The x-axis shows the true biological age in days. While the binarized model stays relatively stable over age, the unbinarized model increases the variance in the prediction error.

**Figure S3. Explanation of the 2nd rescaling**

(A, C, E) Standard lifespan curves of *C. elegans* with a median lifespan of 15.5 days. The X mark the chronological age for which we show the hypothetical age distributions in (B, D, F) respectively. (B, D, F) show the biological age distribution around the chronological age marked by the X. The biggest portion of the age-synchronized worm population will be as old as the chronological age. However, assuming a normal distribution of the biological age, we can assume that a part of the population is biologically younger, respective older. The green lines indicate the median biological age of the living worm population. The dotted line displays the maximum lifespan.

(A, B) All non-censored worms are still alive in the population, i.e. no worm crossed the maximum lifespan line. The population age median is equal to the peak of the distribution.

(C, D) The first (biologically older) worms died, leading to a truncation of the alive distribution of biological age in the population. This has the consequence that the true median of the alive fraction of the worms will be shifted to the left, away from the peak of the distribution.

(E, F) At the median lifespan, 50 % of the population has died. Assuming a uniform shift of the biological age distribution results in the truncation of the right half of the distribution. The true population median is therefore even further shifted to the left.

**Figure S4. Comparison of the model with unbinarized data, random genes and the theoretical limit**

(A) Prediction of the 8 independent datasets consisting of 94 samples at different time points. The x-axis shows the rescaled biological age in days starting from adulthood additionally corrected by the second rescaling approach. The y-axis shows the predicted age computed by an elastic net regression on unbinarized CPMs. For more details on the data see the Table S1. (B) The y-axis shows the mean absolute error (MAE), respective the median absolute deviation (MAD) of a given prediction in days. The box plots display the results of 1000 random models with 576 (binarized) genes. The prediction by our final model with a MAE of 0.45 and a MAD of 0.32 is shown as the blue dots and indicated by arrows. The dotted lines show the theoretical limit of prediction given by the limit of accuracy in the chronological age annotation as well as variance in the lifespan data used for rescaling.

**Figure S5. Comparison of our gene set to published gene sets**

Results of the biological age prediction computed by cross-validation based on different gene sets predicted by Tarkhov et al.(Tarkhov et al. 2019). The x-axis shows the rescaled biological age in days starting from adulthood additionally corrected by the second rescaling approach. The y-axes show the predicted age computed by an elastic net regression on unbinarized (A, B, C) or binarized (D, E, F) gene expression data. Every blue dot displays one RNA-seq sample. The regression lines with the 95 % confidence intervals are shown in blue and the dotted lines show the perfect linear correlation. The distribution of the data is shown on the side of the plot. r²= coefficient of determination, Pearson= Pearson correlation, Spearman= Spearman correlation, MAE= mean absolute error in days, MAD= median absolute deviation in days, RMSE= root-mean-square-error in days.

(A) Prediction based on the unbinarized CPMs of 327 genes generated by a meta-analysis of publicly available microarray data.

(B) Prediction based on the unbinarized CPMs of 902 age-associated genes generated by an RNA-seq experiment.

(C) Prediction based on the unbinarized CPMs of a sparse subset with 71 genes.

(D) Prediction based on the binarized CPMs of the 327 genes generated by a meta-analysis of publicly available microarray data shown in (A).

(E) Prediction based on the binarized CPMs of the 902 age-associated genes generated by an RNA-seq experiment shown in (B).

(F) Prediction based on the binarized CPMs of the sparse subset with 71 genes shown in (C).

**Figure S6. Comparison of our gene set to published gene sets on the validation data**

Prediction of the 8 independent datasets consisting of 94 samples at different time points based on different gene sets predicted by Tarkhov et al.(Tarkhov et al. 2019). The x-axis shows the rescaled biological age in days starting from adulthood additionally corrected by the second rescaling approach. The y-axes show the predicted age computed by an elastic net regression on unbinarized (A, B, C) or binarized (D, E, F) gene expression data. Every blue dot displays one RNA-seq sample. The regression lines with the 95 % confidence intervals are shown in blue and the dotted lines show the perfect linear correlation. The distribution of the data is shown on the side of the plot. r²= coefficient of determination, Pearson= Pearson correlation, Spearman= Spearman correlation, MAE= mean absolute error in days, MAD= median absolute deviation in days, RMSE= root-mean-square-error in days.

(A) Prediction based on the unbinarized CPMs of 327 genes generated by a meta-analysis of publicly available microarray data.

(B) Prediction based on the unbinarized CPMs of 902 age-associated genes generated by an RNA-seq experiment.

(C) Prediction based on the unbinarized CPMs of a sparse subset with 71 genes.

(D) Prediction based on the binarized CPMs of the 327 genes generated by a meta-analysis of publicly available microarray data shown in (A).

(E) Prediction based on the binarized CPMs of the 902 age-associated genes generated by an RNA-seq experiment shown in (B).

(F) Prediction based on the binarized CPMs of the sparse subset with 71 genes shown in (C).

**Figure S7. Biological age prediction of additional samples**

(A) The genotype-dependent effect of dietary restriction (DR) is resembled in the prediction of chronologically 6-day adults. A two-way ANOVA shows a significant interaction effect (p=0.004) between the genotype and the diet. AL = *ad libitum* fed. Data from GSE92909.

(B) The change in diet from K12 to K12*∆tnaA* *E. coli* shows an increasing trend, especially in chronologically older population, as indicated by the different colors. A two-way ANOVA shows a significant diet effect (p=0.03) and almost significant interaction effect (p=0.067). Data from GSE101910.

**Figure S8. Theoretical error in the prediction of the median lifespan from the biological age**

This plot visualizes the intrinsic random error that propagates from the biological age calculation to the fold-change. The x-axis shows the chronological age in days starting from adulthood. The y-axis shows the calculated fold-change between 2 lifespan curves. 3 lifespan comparisons are shown (color-coded). The control median lifespan is always set to 15.5 days, while the second lifespan is variable at 8 days(blue), 15.5 days(orange), and 31 days (green). The same intrinsic biases as in Fig. 2c and Fig. S4b are considered, i.e. a chronological age reporting error of +/- 12 h and a moderate 5 % lifespan variation. For each chronological age point the biological age was calculated with error propagation. The 2 biological age points were then used to approximate the lifespan fold-change for the 3 examples shown. The lines show the average fold-change, e.g. if both lifespans were at 15.5 days (orange), the expected fold-change is at 1.0, i.e. no change. The random error especially introduces a potential bias in the prediction based on chronologically younger samples, i.e. the shadow around the lines.

**Figure S9. Chromosome enrichment**

(A) Chromosome distribution of the 286 protein-coding predictor genes with a coefficient <=0 in blue and the number of protein-coding genes that would be expected if the genes were randomly distributed among the chromosomes in red.

(B) Chromosome distribution of the 260 protein-coding predictor genes with a coefficient >0 in blue and the number of protein-coding genes that would be expected if the genes were randomly distributed among the chromosomes in red.

(C) Differences of the observed to the expected numbers in percent for the protein-coding genes with a coefficient >0 in blue and with a coefficient <=0 in red.

*p<0.05, **p<=0.01, ***p<=0.001, Hypergeometric tests were performed and the resulting p-values were corrected with the Benjamini-Hochberg procedure. Table S3 contains more detailed statistics.

**Figure S10. Motif enrichment nearby the TSS**

Results of a motif enrichment analysis for the region -300 bp to +100 bp from the transcription start site of the genes with a coefficient <=0 (A) and genes with a coefficient >0 (B). The columns show the name of the transcription factor in the first column with the known motif in the second column. Column 3 and 4 show the percentage of target genes, respective background genes, containing the motif in the described region. Column 5 shows the fold change enrichment, column 6 the corresponding Hypergeometric p-value and the last column the Benjamini-Hochberg adjusted q-value.

**Figure S11. Unbinarized human data**

(A) Results of the age prediction computed by cross-validation on human fibroblast gene expression data. The x-axis shows the chronological age in years. The y-axis shows the predicted age computed by an elastic net regression on unbinarized gene expression data. Every blue dot displays one RNA-seq sample. The regression line with the 95 % confidence interval is shown in blue and the dotted line shows the perfect linear correlation. The distribution of the data is shown on the side of the plot. r²= coefficient of determination, Pearson= Pearson correlation, Spearman= Spearman correlation, MAE= mean absolute error in years, MAD= median absolute deviation in years, RMSE= root-mean-square-error in years. Data from GSE113957.

(B) Box plots of age predictions of samples from Hutchinson–Gilford progeria syndrome patients (red) and predictions of age-matched healthy controls (blue) by the elastic net regression of unbinarized gene expression data. Progeria samples show no significant increase in the predicted age compared to age-matched healthy controls. Data from GSE113957.

The p-value was calculated by an independent two-sided t-test. Table S3 contains more detailed statistics.

**Table S1. Data overview**

**Table S2. *C. elegans* age prediction gene set**

**Table S3. Statistics**

**Table S4. Lifespan Prediction**

**Table S5. Human age prediction gene set**
